# Heterophase fcc-2H-fcc gold nanorods

**DOI:** 10.1038/s41467-020-17068-w

**Published:** 2020-07-03

**Authors:** Zhanxi Fan, Michel Bosman, Zhiqi Huang, Ye Chen, Chongyi Ling, Lin Wu, Yuriy A. Akimov, Robert Laskowski, Bo Chen, Peter Ercius, Jian Zhang, Xiaoying Qi, Min Hao Goh, Yiyao Ge, Zhicheng Zhang, Wenxin Niu, Jinlan Wang, Haimei Zheng, Hua Zhang

**Affiliations:** 1 Department of Chemistry, City University of Hong Kong, Hong Kong, China; 2 Hong Kong Branch of National Precious Metals Material Engineering Research Center (NPMM), City University of Hong Kong, Hong Kong, China; 3 Center for Programmable Materials, School of Materials Science and Engineering, Nanyang Technological University, 50 Nanyang Avenue, Singapore, 639798 Singapore; 4 Materials Sciences Division, Lawrence Berkeley National Laboratory, Berkeley, CA 94720 USA; 5 Department of Materials Science and Engineering, National University of Singapore, 9 Engineering Drive 1, Singapore, 117575 Singapore; 6 Institute of Materials Research and Engineering, Agency for Science, Technology, and Research (A*STAR), 2 Fusionopolis Way, Singapore, 138634 Singapore; 7 School of Physics, Southeast University, 211189 Nanjing, China; 8 Institute of High Performance Computing, Agency for Science, Technology, and Research (A*STAR), 1 Fusionopolis Way, #16-16 Connexis, Singapore, 138632 Singapore; 9 National Center for Electron Microscopy, Molecular Foundry, Lawrence Berkeley National Laboratory, Berkeley, CA 94720 USA; 10 Singapore Institute of Manufacturing Technology, Agency for Science, Technology, and Research (A*STAR), 71 Nanyang Drive, Singapore, 638075 Singapore; 11 Department of Materials Science and Engineering, University of California, Berkeley, CA 94720 USA

**Keywords:** Electrocatalysis, Structural properties

## Abstract

The crystal phase-based heterostructures of noble metal nanomaterials are of great research interest for various applications, such as plasmonics and catalysis. However, the synthesis of unusual crystal phases of noble metals still remains a great challenge, making the construction of heterophase noble metal nanostructures difficult. Here, we report a one-pot wet-chemical synthesis of well-defined heterophase fcc-2H-fcc gold nanorods (fcc: face-centred cubic; 2H: hexagonal close-packed with stacking sequence of “AB”) at mild conditions. Single particle-level experiments and theoretical investigations reveal that the heterophase gold nanorods demonstrate a distinct optical property compared to that of the conventional fcc gold nanorods. Moreover, the heterophase gold nanorods possess superior electrocatalytic activity for the carbon dioxide reduction reaction over their fcc counterparts under ambient conditions. First-principles calculations suggest that the boosted catalytic performance stems from the energetically favourable adsorption of reaction intermediates, endowed by the unique heterophase characteristic of gold nanorods.

## Abstract (web summary)

The crystal phase-based heterostructures of noble metal nanomaterials are of interest for various applications. Here, the authors report the wet-chemical synthesis of gold nanorods with a well-defined fcc-2H-fcc heterophase, which possess unique optical and catalytic properties.

## Introduction

Crystal phase control is one of the effective strategies of phase engineering of nanomaterials (PEN)^1^ to modulate the intrinsic physicochemical properties of materials via delicately tuning the order of atom arrangements^2–7^. Although noble metal nanomaterials have been widely investigated for decades, the previous studies almost focus on the size-, dimension-, shape-, architecture-, facet-, lattice strain- and composition-control of thermodynamically stable crystal phase^8–19^. Up to now, it remains difficult to synthesize noble metal nanomaterials with unusual crystal phase compared to their conventional thermodynamically stable phase. Taking gold (Au) as a typical example, although the colloidal synthesis of Au nanoparticles (NPs) dates back to the 1850s^20^, nearly all Au nanostructures synthesized by wet-chemical methods adopt the thermodynamically stable face-centred cubic (fcc) phase. Recently, Au nanomaterials with the unusual hexagonal close-packed (hcp, the 2H type) and 4H phases have been successfully synthesized in solutions^21,22^. However, the high-yield synthesis of 2H and 2H-based Au nanomaterials is yet to be realized, restraining the exploration of their intriguing properties and potential applications.

Owing to their unique optical property, distinct electronic structure, good biocompatibility and extraordinary stability, noble metal nanomaterials possess diverse applications in plasmonics^23^, catalysis^14–16,18,24,25^, biosensing^26^ and in vivo therapy^27^. In particularly, noble metal nanomaterials can be used as electrocatalysts toward carbon dioxide (CO_2_) reduction reaction (CO_2_RR) that converts CO_2_ into valuable chemicals, such as carbon monoxide (CO), ethylene, methane and formic acid^28–32^. Among these products, CO is an important product that can be readily used in downstream Fischer-Tropsch (FT) synthesis for various chemicals and fuels^33^. To date, although great efforts, including size-, composition-, morphology-, surface- and defect-control of noble metal catalysts have been devoted to enhancing CO production from CO_2_RR^34–38^, they still suffer from the low mass activity. Note that the crystal phase of almost all the reported noble metal nanocatalysts for CO_2_RR is limited to their thermodynamically stable crystal phase. On the other hand, crystal phase-based heterostructures have recently attracted increasing research interests because the synergistic effect between two different crystal phases (e.g. phase junction) of inorganic nanomaterials could significantly improve their physicochemical properties, such as optical, electronic and catalytic properties^4,5,39–41^. Unfortunately, it is still a big challenge to synthesize well-defined heterophase noble metal nanomaterials.

Here, we report the direct synthesis of Au nanorods (NRs) with a well-defined fcc-2H-fcc heterophase under mild conditions. By combining experimental and theoretical studies at the single particle level, we identify the different optical properties in fcc, 2H and heterophase fcc-2H-fcc Au NRs. As a proof-of-concept application, we find that the heterophase fcc-2H-fcc Au NRs exhibit much greater electrocatalytic activity compared to the fcc Au NRs and Au NPs in the CO_2_RR. Theoretical calculations reveal that the energetically favourable adsorption of reaction intermediates accounts for the enhanced catalytic performance of heterophase Au NRs.

## Results

### Synthesis and characterization

**Fig. 1:**
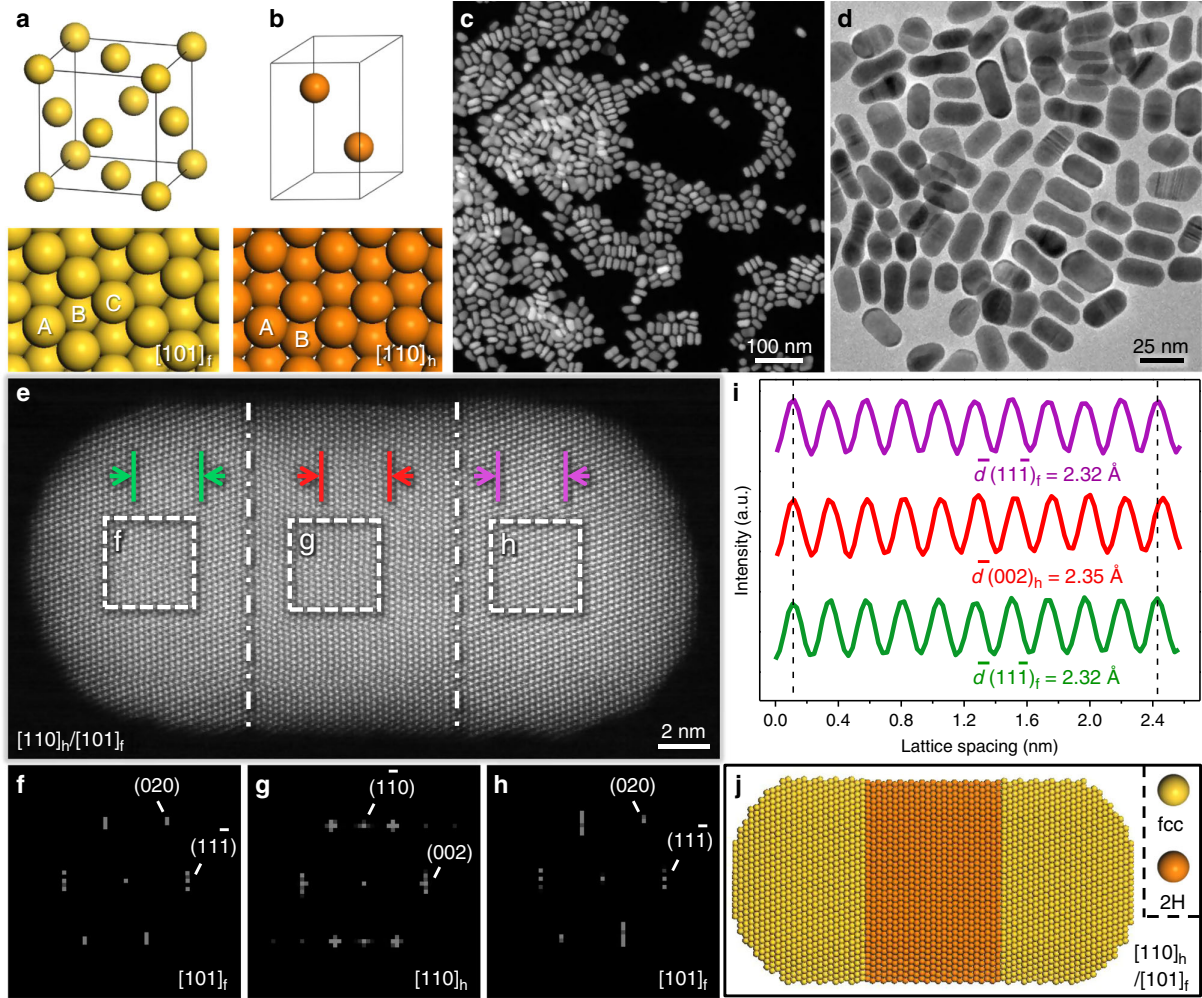
Synthesis and structural characterization of well-defined heterophase fcc-2H-fcc gold nanorods. **a** Schematic illustration of a unit cell (top panel) and (101)_f_ plane (bottom panel) for the conventional fcc phase. **b** Schematic illustration of a unit cell (top panel) and (110)_h_ plane (bottom panel) of the unusual 2H phase. **c**, **d** Low-magnification HAADF-STEM (**c**) and high-magnification TEM (**d**) images of Au NRs. **e** A representative aberration-corrected high-resolution HAADF-STEM image of an Au NR. The fcc/2H phase boundaries are marked with white dashed-dotted lines. **f**–**h** FFT patterns of the corresponding selected areas from the two ends (**f**, **h**) and middle (**g**) of the Au NR shown in **e**. **i** The integrated pixel intensities along the arrow directions of the corresponding selected areas in the two ends (green and purple curves) and middle (red curve) of the Au NR shown in **e**. The peaks and valleys stand for the alternating atoms and spaces, respectively. **j** An atomic model of the obtained heterophase fcc-2H-fcc Au NRs, viewed along the [110]_h_/[101]_f_ zone axes.

The heterophase fcc-2H-fcc Au NRs were synthesized by a one-pot approach in which the potassium tetrachloroaurate was reduced in a mixture of oleylamine and n-dodecylamine at 65 °C (See “Methods” for details). Two crystal phases, i.e. fcc and 2H, of Au are schematically illustrated in Fig. 1a, b, respectively, in which the close-packed planes exhibit different characteristic stacking sequences, i.e. “ABC” for the fcc phase and “AB” for the 2H phase. The X-ray photoelectron spectroscopy (XPS) core level spectrum of the Au 4f doublet confirms the metallic state of Au in the fcc-2H-fcc Au NRs (Supplementary Fig. 1). The transmission electron microscopy (TEM) and high-angle annular dark-field scanning TEM (HAADF-STEM) images reveal the size distribution of the obtained Au NRs (Fig. 1c, d), i.e. the aspect ratio is ~2, with a length of 28.2 ± 5.7 nm and a width of 14.6 ± 3.2 nm (Supplementary Fig. 2). It is worth mentioning that the correct choice of Au precursor (Supplementary Fig. 3 and Supplementary Note 1), solvent (Supplementary Fig. 4 and Supplementary Note 2) and gas atmosphere (Supplementary Fig. 5 and Supplementary Note 3) in the reaction is important for the successful synthesis of heterophase fcc-2H-fcc Au NRs.

The crystal phase of as-prepared Au NRs was examined with aberration-corrected HAADF-STEM. Figure 1e shows a representative aberration-corrected high-resolution HAADF-STEM image of an Au NR, which exhibits distinct types of atom arrangements along its long axis. Selected-area fast Fourier transform (FFT) patterns from the two ends of Au NRs match well with the typical [101]_f_-zone axis diffraction pattern of fcc phase, exhibiting the diffraction spots of (020)_f_ and (11$\bar 1$)_f_ planes (Fig. 1f, h). In contrast, the selected-area FFT pattern from the middle of Au NRs is consistent with the characteristic [110]_h_-zone axis diffraction pattern of the 2H phase, demonstrating the diffraction spots of the (1$\bar 1$0)_h_ and (002)_h_ planes (Fig. 1g). As indicated by the dashed-dotted white lines in Fig. 1e, there are sharp interfaces between two different phases, and a well-defined sandwich structure of fcc-2H-fcc heterophase along the close-packed directions of [111]_f_/[001]_h_ is formed. Figure 1i shows the integrated pixel intensities of 2H (002)_h_ and fcc (11$\bar 1$)_f_ lattices from the selected areas in Fig. 1e. The average interlayer spacing of (002)_h_ planes was calculated to be 2.35 Å (red curve), which is slightly (ca. 1.3%) greater than that of (11$\bar 1$)_f_ planes (2.32 Å, green and purple curves). Based on the aforementioned result, an atomic model of the as-prepared heterophase fcc-2H-fcc Au NRs is schematically illustrated in Fig. 1j.

To further corroborate the heterophase structure in the synthesized fcc-2H-fcc Au NRs, high-resolution TEM (HRTEM) images from the other two directions, i.e. Y direction and Z direction (Supplementary Fig. 6), were collected. The corresponding FFT pattern of HRTEM image collected from the Y direction agrees well with the diffraction patterns of [1$\bar 1$0]_h_ and [$\bar 1\bar 2$1]_f_ zone axes (Supplementary Fig. 7). From the corresponding FFT pattern of the HRTEM image taken from the Z direction (i.e. along the [001]_h_/[11$\bar 1$]_f_ zone axes), two sets of diffraction spots belonging to (1$\bar 1$0)_h_ and (202)_f_ planes, respectively, can be distinguished (Supplementary Fig. 8), providing further evidence for the unique fcc-2H-fcc heterophase structure of Au NRs. Besides, the structure of fcc-2H-fcc Au NRs was also confirmed by the X-ray diffraction (XRD) pattern, which demonstrates two sets of diffraction peaks belonging to 2H and fcc phases (Supplementary Fig. 9).

### Optical property

**Fig. 2:**
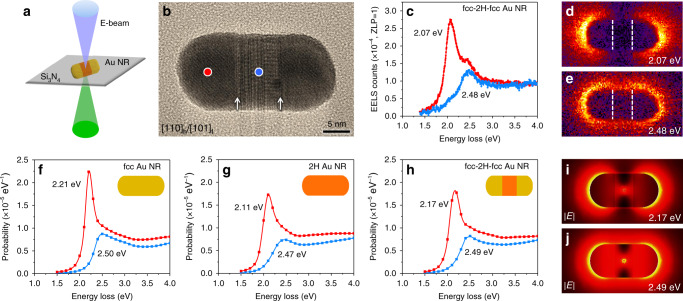
Monochromated EELS analysis of an individual heterophase fcc-2H-fcc gold nanorod. **a** Schematic illustration of the monochromated EELS measurement: an electron probe with beam size of ~1 nm^2^ is raster-scanned in a rectangular region around the fcc-2H-fcc Au NR, while a monochromated EELS spectrum is measured in each scan pixel. **b** HRTEM image of an individual fcc-2H-fcc Au NR. The fcc/2H phase boundaries are indicated by white arrows. **c** Experimental EELS spectra collected in the end (red curve) and middle (blue curve) of fcc-2H-fcc Au NR, as indicated by the red and blue dots in **b**, respectively. **d**, **e** Measured EELS maps of the fcc-2H-fcc Au NR shown in **b**, displaying the intensity EELS distribution at 2.07 eV (**d**) and 2.48 eV (**e**). The positions of fcc/2H phase boundaries are marked by white dashed lines. **f**–**h** Calculated EELS spectra for fcc Au NR (**f**), 2H Au NR (**g**) and fcc-2H-fcc Au NR (**h**) at two E-beam excitation locations: in the end (red curve) and middle (blue curve). Insets: the corresponding theoretical models of single fcc Au NR (**f**), 2H Au NR (**g**) and fcc-2H-fcc Au NR (**h**). **i**, **j** Simulated total field distributions at two resonant energies of 2.17 eV (**i**) and 2.49 eV (**j**) excited by an E-beam in the middle of an fcc-2H-fcc Au NR.

Monochromated electron energy loss spectroscopy (EELS) was used to investigate the electromagnetic eigenmodes of fcc-2H-fcc Au NRs at the single particle level (Fig. 2a, see “Methods” for details). The monochromated EELS experiments were conducted by raster-scanning an electron probe with beam size of ~1 nm^2^ within a rectangular area containing an individual fcc-2H-fcc Au NR. Figure 2b shows the HRTEM image of a fcc-2H-fcc Au NR with length, width and aspect ratio of 29.8 nm, 14.6 nm and 2.0, respectively. The corresponding EELS spectra of a single fcc-2H-fcc Au NR were collected from different locations, as marked in Fig. 2b. Two distinct eigenmode resonances were observed with one at 2.48 eV and the other at 2.07 eV excited by placing the electron beam (E-beam) in the middle and end of fcc-2H-fcc Au NR, respectively (Fig. 2c). In addition, the monochromated EELS maps demonstrate that the high-energy eigenmode is distributed over the whole surface of fcc-2H-fcc Au NR, while the low-energy eigenmode is excited at the two ends (Fig. 2d, e).

In order to reveal the effect of crystal phase on the electromagnetic eigenmodes of Au NRs, density functional theory (DFT) computation (Supplementary Fig. 10) and three-dimensional electromagnetic full-wave EELS simulation were used to calculate the EELS spectra (see “Methods” for details). Figure 2f–h show the calculated EELS spectra for three different types of Au NRs, i.e. pure fcc Au, pure 2H Au, and heterophase fcc-2H-fcc Au, as schematically illustrated in the insets. To mimic the EELS experiment, we excite Au NRs with the E-beam at two different locations. A low-energy peak is dominant in the end (red curve) of the Au NR, while a high-energy peak is more prominent in its middle (blue curve), consistent with the experimental observation (Fig. 2c). In comparison with the pure fcc Au NR (Fig. 2f), the optical responses of pure 2H and heterophase fcc-2H-fcc Au NRs exhibit both position shifts and intensity variations for all the eigenmode resonances in the range of 1.5–4.0 eV (Fig. 2g, h). To uncover the nature of the eigenmodes associated with the two peaks of fcc-2H-fcc Au NRs, we performed a Helmholtz decomposition analysis to reveal the contributions of the transverse and longitudinal fields. The peaks observed in the EELS probability plot (Supplementary Fig. 11) are confirmed to be resonances on surface plasmon polaritons. The fields, excited by the E-beam located in the middle of fcc-2H-fcc Au NRs, are shown in Fig. 2i, j. The electric field is concentrated at two ends for the low-energy mode (Fig. 2i), while for the high-energy mode, it distributes over the entire particle (Fig. 2j), agreeing well with the experimental results (Fig. 2d, e).

### Catalytic property

**Fig. 3:**
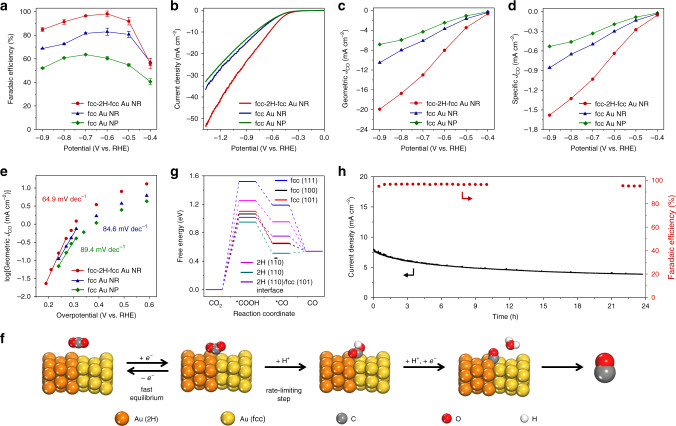
Electrocatalytic performance of heterophase fcc-2H-fcc gold nanorods in carbon dioxide reduction reaction. **a** The CO Faradaic efficiency of fcc-2H-fcc Au NRs, fcc Au NRs and fcc Au NPs at different potentials. **b** LSV curves recorded in CO_2_-saturated aqueous solution of 0.5 M KHCO_3_ with a scan rate of 10 mV s^−1^. **c**, **d** Partial current densities normalized by the geometric surface area of carbon electrodes (**c**) and ECSA of different Au nanostructures (**d**). **e** Tafel plots of fcc-2H-fcc Au NRs, fcc Au NRs and fcc Au NPs. **f** Schematic illustration for the CO_2_RR pathway on the regional surface of fcc-2H-fcc Au NRs. **g** Free energy diagrams for CO_2_ reduction via the formation of *COOH to produce CO on different surfaces, including the 2H (110), 2H (110)/fcc (101) interface, 2H (1$\bar 1$0), fcc (100), fcc (101) and fcc (111). **h** Electrocatalytic stability test of fcc-2H-fcc Au NRs at applied potential of −0.6 V (vs. RHE). The CO Faradaic efficiency was analysed every half an hour in the first 10 h and the last 2 h as well.

It is well recognized that the surface property of noble metal catalysts plays a key role in dictating their catalytic performance^42,43^. In electrochemical CO_2_RR, the size^31^, shape^44^, grain boundary^37,45^ and surface ligand^46^ of catalysts have been identified as key factors that can significantly alter their surface properties, and hence their catalytic performance. These studies provide deeper understanding of the CO_2_RR mechanism, which in turn promotes the development of CO_2_ reduction technology. Here, by using the synthesized unique heterophase fcc-2H-fcc Au NR as a new model catalyst, we explore the critical role of the unusual 2H phase as well as the interface between 2H and fcc phases in CO_2_RR. We investigated the CO_2_RR performance of fcc-2H-fcc Au NRs by using the H-type cell with three-electrode system under ambient conditions and analysed the gaseous products with an on-line gas chromatography (See “Methods” for details). For comparison, fcc Au NRs and fcc Au NPs were synthesized (Supplementary Figs. 12 and 13) and used for the CO_2_RR test under the same experimental conditions. All catalysts were thoroughly washed to remove their surface capping ligands (Supplementary Fig. 14). As shown in Fig. 3a, fcc-2H-fcc Au NRs exhibited over 90% selectivity towards CO production from −0.5 to −0.8 V (vs. RHE) and reached the highest Faradaic efficiency (FE) of 98.2% at −0.6 V (vs. RHE). The competing hydrogen evolution reaction was almost entirely suppressed (Supplementary Fig. 15). In contrast, the highest FE for fcc Au NRs and fcc Au NPs were only 81.6% and 63.6%, respectively. In addition, the linear sweep voltammetry (LSV) measurements showed that fcc-2H-fcc Au NRs have lower onset potential and much higher current density compared to fcc Au NRs and fcc Au NPs (Fig. 3b). As a result, fcc-2H-fcc Au NRs showed greater CO partial current density (J_CO_) in a wide range of potentials (Fig. 3c). At the applied potential of −0.6 V (vs. RHE), J_CO_ reached −8.03 mA cm^−2^, which was 2.2 and 3.2 times of that of fcc Au NR and fcc Au NP, respectively. These results suggest that the heterophase fcc-2H-fcc Au nanorods possess superior catalytic selectivity and activity in CO_2_RR, compared with their fcc counterparts. Notably, the mass activity at the potential of −0.6 V (vs. RHE) reached 30.8 A g^−1^ (Supplementary Fig. 16), which is higher compared to the previously reported noble metal catalysts (Supplementary Table 1).

To gain insight into the origin of the superior CO_2_RR performance of fcc-2H-fcc Au NRs, we first measured the specific activity by normalizing the current density to the electrochemically active surface area (ECSA). Note that ECSAs of the three Au catalysts were estimated by underpotential deposition of copper on their surfaces (Supplementary Fig. 17). As shown in Fig. 3d, fcc-2H-fcc Au NRs showed much higher specific activity than that of their fcc counterparts. In specific, at the applied potential of −0.6 V (vs. RHE), fcc-2H-fcc Au NR demonstrated a high specific activity of −0.64 mA cm^−2^, which is 2.1 and 3.4 times that of fcc Au NR and fcc Au NP, respectively. These results indicate that the intrinsic catalytic activity of the heterophase fcc-2H-fcc Au NRs is much higher than that of their fcc counterparts.

As shown in Fig. 3e, based on the plot of the logarithm of geometric J_CO_ versus overpotential, the Tafel slope was calculated by linear fitting at low overpotentials. As a result, the heterophase fcc-2H-fcc Au NR exhibited a much smaller Tafel slope of 64.9 mV dec^−1^, compared to the fcc Au NR (84.6 mV dec^−1^) and fcc Au NP (89.4 mV dec^−1^). The values of these Tafel slopes revealed that all three Au catalysts shared a common reaction mechanism. It means that a fast one-electron transfer to the adsorbed CO_2_ to generate the surface-adsorbed CO_2_^∙−^ occurs first, and the subsequent combination of CO_2_^∙−^ and a proton to form *COOH is the rate-limiting step (Fig. 3f)^30,46^. Therefore, the smaller Tafel slope of fcc-2H-fcc Au NR suggests a faster electron exchange rate (i.e. faster reaction kinetics).

First-principles calculations were conducted to further elucidate the reaction mechanism. Free energy diagrams for CO_2_RR towards the CO production were calculated for the three Au catalysts. The calculation models are shown in Supplementary Fig. 18. Consistent with the Tafel analysis (Fig. 3e), the first-step reduction of CO_2_ into *COOH is the rate-limiting step, as verified by the positive reaction free energy (ΔG_*COOH_) for all studied surfaces (Fig. 3g). Note that the values of ΔG_*COOH_ for 2H (110), 2H (110)/fcc (101) interface, 2H (1$\bar 1$0), fcc (100), fcc (101) and fcc (111) are 0.95, 1.02, 1.25, 1.06, 1.10 and 1.52 eV, respectively. In the next step, the produced *COOH reacts with a proton/electron pair to produce *CO and H_2_O, which was found to be exothermic. In general, 2H (110) surface and 2H (110)/fcc (101) interface possess lower ΔG_*COOH_ compared to the representative fcc Au surfaces, indicating a lower kinetic barrier for CO_2_RR. Therefore, the excellent CO_2_RR catalytic performance of the heterophase fcc-2H-fcc Au NRs could be attributed to the presence of unusual 2H phase and the interface between 2H and fcc phases.

A long-term durability test was carried out to evaluate the stability of fcc-2H-fcc Au NRs towards CO_2_RR. Notably, at the applied potential of −0.6 V (vs. RHE), the FE for CO production showed negligible decrease, and was maintained above 95% after an almost 24-h test (Fig. 3h). Meanwhile, the structure characterization of Au NRs after the durability test identified that the fcc-2H-fcc heterophase of Au NRs was well preserved, suggesting that the heterophase Au NRs possess outstanding electrochemical stability (Supplementary Fig. 19).

## Discussion

In summary, we have realized a one-pot synthesis of well-defined heterophase fcc-2H-fcc Au NRs. This method overcomes the limitation of the classical seeded growth approach, which usually generates Au NRs with the thermodynamically stable fcc phase^47–49^. Compared with the common fcc Au NRs, the heterophase fcc-2H-fcc Au NRs demonstrate novel optical features like the energy shift and intensity modulation of surface plasmon polariton resonances. Furthermore, the electrocatalytic activity of heterophase fcc-2H-fcc Au NRs toward CO_2_RR outperforms their fcc counterparts due to the energetically more favourable adsorption of reaction intermediates on the 2H-phase surface and the heterophase 2H/fcc interface, which was confirmed by first-principles calculations. It is believed that the controlled synthesis of well-defined heterophase nanostructures not only provides an effective strategy to fine-tune the physicochemical properties and catalytic performances of noble metal nanomaterials, but also opens up an avenue for fundamentally understanding the crystallization behaviour of noble metals at the nanometer scale.

## Methods

### Materials

Gold(III) chloride hydrate (HAuCl_4_∙aq, 52% Au basis), potassium tetrachloroaurate (KAuCl_4_, 99.995%), silver nitrate (AgNO_3_, ACS reagent, ≥99.0%), oleylamine (70%, technical grade), n-dodecylamine (98%), cyclohexane (anhydrous, 99.5%), H_2_SO_4_ (98%), L-ascorbic acid (crystalline, ≥99.0%), hexadecyltrimethylammonium bromide (CTAB, ≥99.0%), Nafion solution (5%), potassium bicarbonate (KHCO_3_, ≥99.0%) and the other chemicals without special mention were purchased from Sigma-Aldrich. Ethanol (absolute, 99.9%) and concentrated hydrochloric acid (HCl, 37%) were purchased from Merck. Sodium borohydride (NaBH_4_, ≥99.0%) was purchased from Fluka. Carbon paper (Toray TGP-H-090) was purchased from Fuel Cell Earth. Ultrapure Milli-Q water (Milli-Q System, Millipore, Billerica, MA, USA) was used in the experiments. All the chemicals were used as received without any purification.

### Synthesis of heterophase fcc-2H-fcc Au NRs

In a typical synthesis, 960 mg of n-dodeylamine were first dissolved into 0.8 mL of oleylamine in a glass vial to form a homogeneous solution, denoted as Solution A. Then 3.0 mg of potassium tetrachloroaurate were added into the glass vial containing Solution A, followed by purging oxygen gas into the solution for about 5 min. After the glass vial was sealed, the mixture was sonicated for about 2 min. The resultant homogenous growth solution was then heated in an oil bath at 65 °C for 17 h. After the reaction was completed, 2 mL of the mixture of cyclohexane and ethanol (v/v = 5/1) were added into the resulted solution, followed by sonication for about 1 min. The colloidal product was collected by centrifugation at 7,000 r.p.m. for 3 min, thoroughly washed with a solvent mixture of cyclohexane/ethanol (v/v = 5/1), and then re-dispersed into cyclohexane.

### Synthesis of fcc Au NRs

The fcc Au NRs were synthesized according to a previously reported seeded growth method with a slight modification^50^. First, Au seeds were prepared as follows. Typically, 125 µL of HAuCl_4_ (10 mM, H_2_O) were mixed with 5 mL of CTAB (100 mM, H_2_O) to form a homogeneous solution, denoted as Solution B. Then 300 µL of freshly prepared ice-cold NaBH_4_ (10 mM, H_2_O) were added into the Solution B under vigorous stirring (800 r.p.m.). The resultant seed solution was aged for 20 min with continuous stirring under room temperature prior to use. After that, the Au seeds were used for the growth of Au NRs as follows. In a typical experiment, 0.5 mL of HAuCl_4_ (10 mM, H_2_O) were mixed with 10 mL of CTAB (100 mM, H_2_O) to form a homogeneous solution, denoted as Solution C. Then 200 µL of HCl (1 M, H_2_O), 80 µL of L-ascorbic acid (100 mM, H_2_O) and 15 µL of the freshly prepared Au seed solution were successively added into the Solution C. The resultant growth solution was kept at 30 °C for 1.5 h. After the reaction was completed, the colloidal product was collected by centrifugation at 14,000 r.p.m. for 5 min, thoroughly washed with water, and then re-dispersed into water.

### Synthesis of fcc Au NPs

In a typical experiment, 6.12 mg of HAuCl_4_, 330 µL of oleylamine and 5.31 mL of hexane were successively added in a glass vial. Then the glass vial was carefully sealed and gently shaken to dissolve HAuCl_4_ in the mixture of hexane and oleylamine. The resultant growth solution in the glass vial was heated at 58 °C for 16 h. After the reaction was completed, the colloidal product was collected by centrifugation at 14,500 r.p.m. for 5 min, thoroughly washed with hexane, and then re-dispersed into cyclohexane.

### Eigenmode characterization of individual heterophase fcc-2H-fcc Au NR

HRTEM images of individual fcc-2H-fcc Au NR were taken with a FEI Titan TEM with Schottky electron source, operated at 200 kV. The same instrument in STEM mode was used at 80 kV for the eigenmode characterization. A Wien-type monochromator dispersed the electron beam in energy, and a narrow energy-selecting slit formed a monochrome electron beam with typical full-width at half maximum values of ~80 meV. Using a convergence semi-angle of 13 mrad, a ~1 nm^2^ electron probe was formed and scanned in rectangular areas of pixels (for mapping), or placed at specific locations to acquire individual spectra using a Gatan Tridiem ER EELS detector. All spectra were normalized at their maximum, i.e., the top of zero-loss peak (ZLP). The spectral background signal was corrected by fitting and subtracting a high-quality background spectrum that was measured elsewhere on the same sample without particles nearby.

### DFT computation

The dielectric permittivities of fcc Au and 2H Au were computed using standard DFT approaches, i.e., independent particle approximation and the dipole-transition limit as implemented in density functional theory^51,52^ WIEN2k package (see www.wien2k.at for details). In order to choose the density functional, we have benchmarked the computed dielectric permittivity for bulk fcc Au against measured permittivity in the low-energy region up to 4 eV. It turned out that the modified Becke-Johnson (MBJ)^53^ exchange correlated potential developed by Tran and Blaha^54^ in their latest parametrization^55^ gives the closest match. This exchange correlation potential was subsequently applied to compute the response for Au in the 2H phase.

### EELS simulation

The electron beam in EELS simulations was modelled as a monoenergetic flow of relativistic particles moving with constant velocity. This model is validated^56^ by the average energy loss being negligible compared to the initial energy of electrons (80 keV), as well as by a small change of the beam momentum during its interaction with a thin sample. The electric **E**(ω, **r**) and magnetic **H**(ω, **r**) fields excited by the electron beam at frequency ω were numerically computed in COMSOL Multiphysics following the macroscopic Maxwell’s equations,

1 $$\nabla \times {\mathbf{H}}\left( {\omega ,{\mathbf{r}}} \right) = - i\omega \varepsilon _0\varepsilon \left( {\omega ,{\mathbf{r}}} \right){\mathbf{E}}\left( {\omega ,{\mathbf{r}}} \right) + {\mathbf{J}}\left( {\omega ,{\mathbf{r}}} \right)$$

2 $$\nabla \times {\mathbf{E}}\left( {\omega ,{\mathbf{r}}} \right) = i\omega \mu _0{\mathbf{H}}\left( {\omega ,{\mathbf{r}}} \right)$$

where $\varepsilon _0$ and *μ*_0_ are electric and magnetic constants, respectively, and **J**(*ω*, **r**) is the current density of the electron beam. The space-dependent dielectric permittivity $\varepsilon \left( {\omega ,{\mathbf{r}}} \right)$ was composed from the DFT-derived complex permittivities depending on the nanorod composition. Finally, the energy loss probability was classically computed though the ohmic loss^57^ as follows,

3 $$\Gamma (\omega ) = \frac{1}{{2{\hbar} \omega }}\int_{V} {{\it{Re}}}\left[ {\mathbf{J}}^{\ast} \left( {\omega} ,{\mathbf{r}} \right) \cdot {\mathbf{E}}\left( {\omega ,{\mathbf{r}}} \right) \right]d{\mathbf{r}} $$

where the integration is performed over a volume of the electron beam (*V*).

### Preparation of working electrodes

Typically, 6 mg of Vulcan XC-72R carbon black were mixed with 1 mL of catalyst solution, and the obtained solution was sonicated for 60 min. The solid was collected by centrifuging at 14,000 r.p.m. for 10 min, and re-dispersed into 1 mL of ethanol. Subsequently, 20 µL of Nafion solution were added into the solution containing catalyst and carbon black, and then the mixed solution was sonicated for another 15 min to form a well-dispersed catalyst ink. After that, 100 µL of the resultant catalyst ink were dropped onto both sides of a carbon paper (1.0 cm × 0.5 cm) with a working area of 1.0 cm^2^. The loading amount of Au catalysts was 266 µg. The obtained electrode was dried at room temperature.

### Electrochemical CO_2_RR

CO_2_RR was performed in a gas-tight two-compartment H-type cell separated by an ion exchange membrane (Nafion 117). The electrolyte was CO_2_-saturated 0.5 M KHCO_3_. The H-type cell was connected to an electrochemical workstation (CHI 760E) using a three-electrode system. Platinum mesh and Ag/AgCl (with saturated KCl as the filling solution) were used as counter electrode and reference electrode, respectively. All electrode potentials were converted to the reversible hydrogen electrode (RHE) reference scale using the following equation:

4 $${\mathrm{E}}\left( {{\mathrm{vs}}.\,{\mathrm{RHE}}} \right) = {\mathrm{E}}\left( {{\mathrm{vs}}.\,{\mathrm{Ag}}/{\mathrm{AgCl}}} \right) + 0.197\,{\mathrm{V}} + 0.0591 \times {\mathrm{pH}}$$

Before the test, the electrolyte in the cathodic compartment was bubbled with CO_2_ gas for 30 min. During the test, the electrolyte in the cathodic compartment was stirred at 450 r.p.m. CO_2_ gas was bubbled into the cathodic compartment at a rate of 20.0 standard cubic centimeters per minute (s.c.c.m.), and was routed into an on-line gas chromatograph (GC, Agilent 7890B). The GC analysis was set up to split the gas sample into three aliquots. One aliquot passed a thermal conductivity detector (TCD), and the other two aliquots were routed through two different flame ionization detectors (FIDs).

### FE calculation

The FE and partial current density of CO were calculated from GC chromatogram peak areas, where C_CO_ is the volume concentration of CO based on the calibration of the GC. The equation is shown as follows.

5 $${\mathrm{FE}} = \frac{{C_{{\mathrm{CO}}} \times v \times \frac{{2{\mathrm{F}}p_0}}{{{\mathrm{RT}}}}}}{{i_{{\mathrm{total}}}}} \times 100$$

where *i*_total_ is the measured current, *v* is CO_2_ flow rate, F is Faradaic constant, *p*_0_ is pressure (1.013 bar), *T* is temperature (300 K), and R is the ideal gas constant (8.314 J mol K^−1^).

### ECSA measurements

The ECSA of all the Au nanostructures were measured based on the charge associated with the stripping of an underpotential-deposited Cu monolayer. Briefly, after the working electrode was immersed into N_2_-saturated 0.5 M H_2_SO_4_ solution, cyclic voltammograms (CVs) from 0.05 V to 0.50 V (vs. Ag/AgCl) at a scan rate of 10 mV s^−1^ were acquired repeatedly until traces converged. Subsequently, the working electrode was immersed into a N_2_-saturated 0.5 M H_2_SO_4_ solution containing 0.1 M CuSO_4_. Note that the electrolyte was continuously purged with N_2_. CVs were recorded from 0.05 to 0.50 V (vs. Ag/AgCl) at a scan rate of 10 mV s^−1^. The anodic stripping waves were integrated. The factor used to convert the stripping charge to surface area was 0.42 mC cm^−2^.

### First-principles calculations

All the calculations were performed by using Vienna ab initio simulation package (VASP)^58,59^. The ion-electron interactions were described by the projector augmented wave (PAW) method^60^. The generalized gradient approximation in the revised Perdew-Burke-Ernzerhof (RPBE) functional and a cut-off energy of 400 eV for plane-wave basis set were adopted^61^. The convergence threshold was 10^−5^ eV and 0.03 eV/Å for energy and force, respectively. To avoid the interaction between two periodic units, a vacuum space of 20 Å was used. The models were constructed from four layers of the corresponding Au facets, where the bottom two layers were fixed. The Brillouin zones were sampled by a Monkhorst-Pack k-point mesh with a 3 × 3 × 1 k-point grid. The free energy change (Δ*G*) of each elementary reaction was calculated as follows.

6 $$\Delta G = \Delta E + \Delta E_{{\mathrm{ZPE}}} - T\Delta S + {\int} {C_PdT} + \Delta G_{{\mathrm{sol}}}$$

where *E*, *E*_ZPE_, *T*, *S*, *C*_*P*_ and *G*_sol_ are the electronic energy, zero-point energy, temperature, entropy, heat capacity and correction to the solvation effect on the free energy, respectively. All the corrections we used in this work were summarized in Supplementary Table 2.

### Characterization

The TEM samples were prepared by directly dropping the suspension of different samples onto the full carbon-coated copper grids (200 mesh), followed by drying at ambient conditions. The TEM, HRTEM and HAADF-STEM images were taken on a JEOL JEM-2100F TEM operated at 200 kV. The aberration-corrected HAADF-STEM images were obtained by a JEOL ARM200F (JEOL, Tokyo, Japan) aberration-corrected transmission electron microscope operated at 200 kV with a cold field emission gun and double hexapole C_s_ correctors (CEOS GmbH, Heidelberg, Germany). The XPS samples were prepared by directly dropping the suspension of different samples onto the Si substrates, followed by drying at ambient conditions. The XPS measurements were carried out on a VG ESCALAB 220i-XL instrument (base pressure <10^−5^ mbar) equipped with a monochromatic Al Kα (1486.7 eV) X-ray source. The XRD characterization was conducted on an X-ray diffractometer (Shimadzu, XRD-6000) operated at 30 mA and 40 kV. The concentration of catalyst was estimated by the inductively coupled plasma-optical emission spectroscopy (ICP-OES, Dual-view Optima 5300 DV). The catalysts after the durability measurements were collected from the electrodes with sonication in ethanol and then used for the TEM characterization.

## Supplementary information

Supplementary Information

## Data Availability

The data that support the findings of this study are available from the corresponding author upon reasonable request.
