# Supplementary material for: Heterophase fcc-2H-fcc gold nanorods
Source: Nat Commun. 2020 Jul 3;11:3293. doi: 10.1038/s41467-020-17068-w (PMC7335101; doi:10.1038/s41467-020-17068-w)
Supplement: Supplementary file 1 — Supplementary Information [file 41467_2020_17068_MOESM1_ESM.pdf]

Supplementary Information

Heterophase fcc-2H-fcc gold nanorods

Zhanxi Fan[1,2,3,4†], Michel Bosman[5,6†], Zhiqi Huang[1†], Ye Chen[3†], Chongyi Ling[1,7†], Lin Wu[8], Yuriy A. Akimov[8], Robert Laskowski[8], Bo Chen[3], Peter Ercius[9], Jian Zhang[3], Xiaoying Qi[10], Min Hao Goh[10], Yiyao Ge[3], Zhicheng Zhang[3], Wenxin Niu[3], Jinlan Wang[7], Haimei Zheng[4,11*], Hua Zhang[1,2*]

[1]Department of Chemistry, City University of Hong Kong, Hong Kong, China

[2]Hong Kong Branch of National Precious Metals Material Engineering Research Center (NPMM), City University of Hong Kong, Hong Kong, China.

[3]Center for Programmable Materials, School of Materials Science and Engineering, Nanyang Technological University, 50 Nanyang Avenue, Singapore 639798, Singapore

[4]Materials Sciences Division, Lawrence Berkeley National Laboratory, Berkeley, California 94720, United States

[5]Department of Materials Science and Engineering, National University of Singapore, 9 Engineering Drive 1, Singapore 117575, Singapore

[6]Institute of Materials Research and Engineering, Agency for Science, Technology, and Research (A*STAR), 2 Fusionopolis Way, Singapore 138634, Singapore

[7]School of Physics, Southeast University, Nanjing 211189, China

[8]Institute of High Performance Computing, Agency for Science, Technology, and Research (A*STAR), 1 Fusionopolis Way, #16-16 Connexis, Singapore 138632, Singapore

[9]National Center for Electron Microscopy, Molecular Foundry, Lawrence Berkeley National Laboratory, Berkeley, California 94720, United States

[10]Singapore Institute of Manufacturing Technology, Agency for Science, Technology, and Research (A*STAR), 71 Nanyang Drive, Singapore 638075, Singapore

[11]Department of Materials Science and Engineering, University of California, Berkeley, California 94720, United States

[†]These authors contributed equally to this work

[*]Corresponding author

E-mail: hua.zhang@cityu.edu.hk; hmzheng@lbl.gov

Supplementary Figures

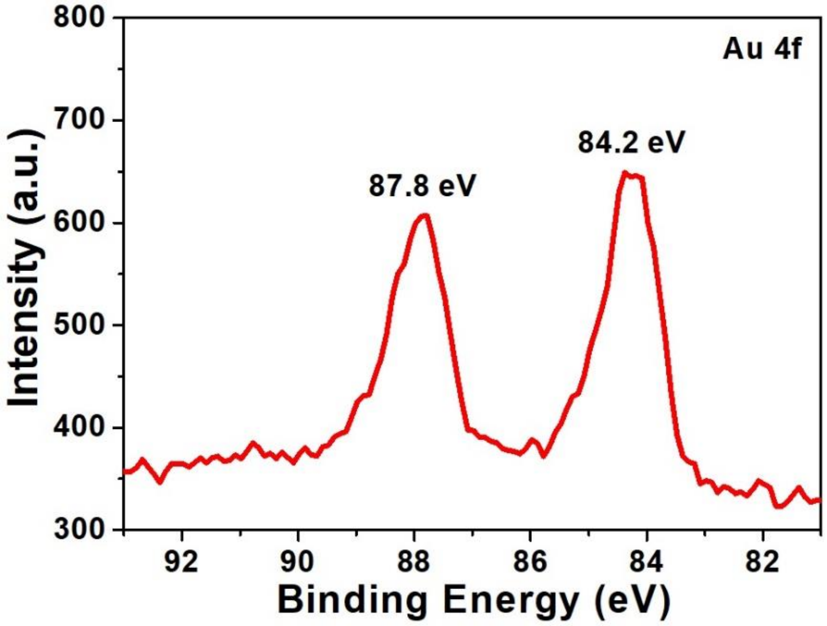

Supplementary Figure 1 | XPS analysis of Au chemical state. The XPS spectrum of fcc-2H-fcc Au NRs, showing the core level peaks of Au 4f doublet. The position of Au 4f doublet identifies the metallic state of Au in the obtained fcc-2H-fcc Au NRs.

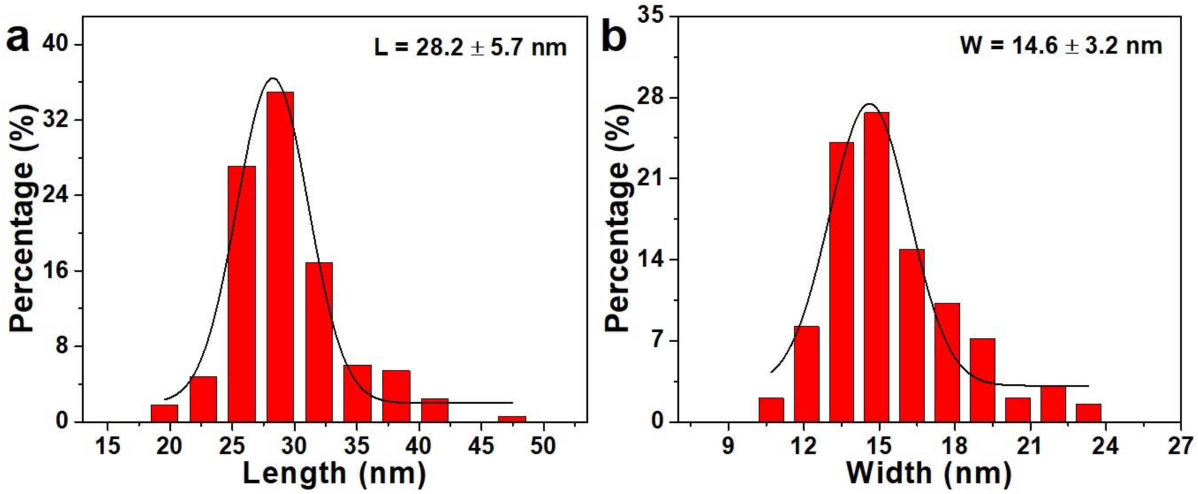

Supplementary Figure 2 | Size distribution analysis of fcc-2H-fcc Au NRs. a, Statistical analysis of the length of Au NRs measured by TEM, in which 28.2 nm is the average length and 5.7 nm is the standard deviation. **b**, Statistical analysis of the width of Au NRs measured by TEM, in which 14.6 nm is the average width and 3.2 nm is the standard deviation. The curves in **a** and **b** were obtained by fitting the data using a Gaussian function.

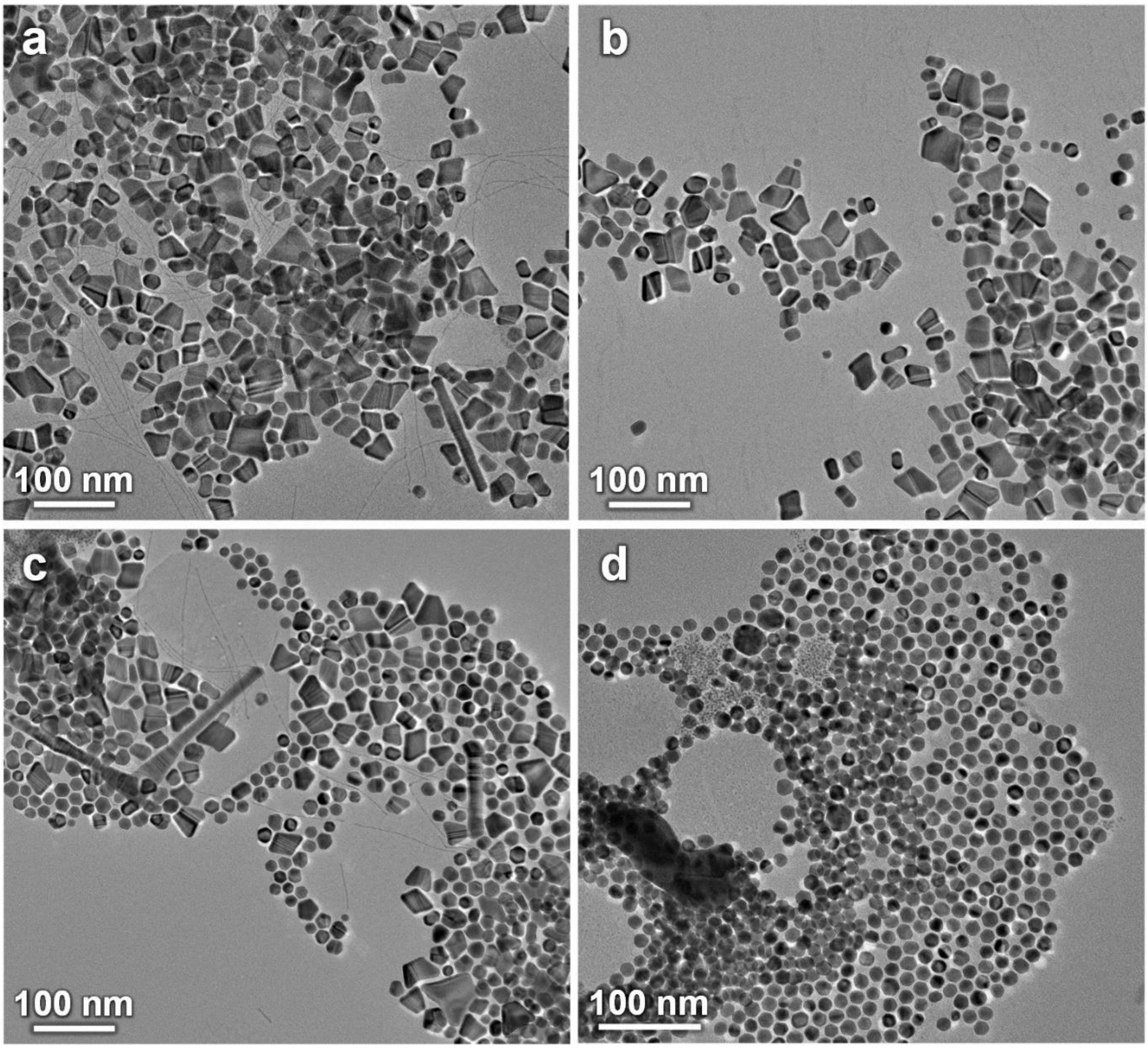

Supplementary Figure 3 | The effect of Au precursors. a-d, TEM images of Au nanostructures obtained by replacing the potassium gold(III) chloride ($KAuCl_4$) with other Au precursors, i.e., (**a**) tetrachloroauric(III) acid ($HAuCl_4$), (**b**) trichloro(pyridine)gold(III) ($AuCl_3 \cdot Py$), (**c**) gold(III) chloride ($AuCl_3$), and (**d**) gold(III) acetate ($Au(ac)_3$).

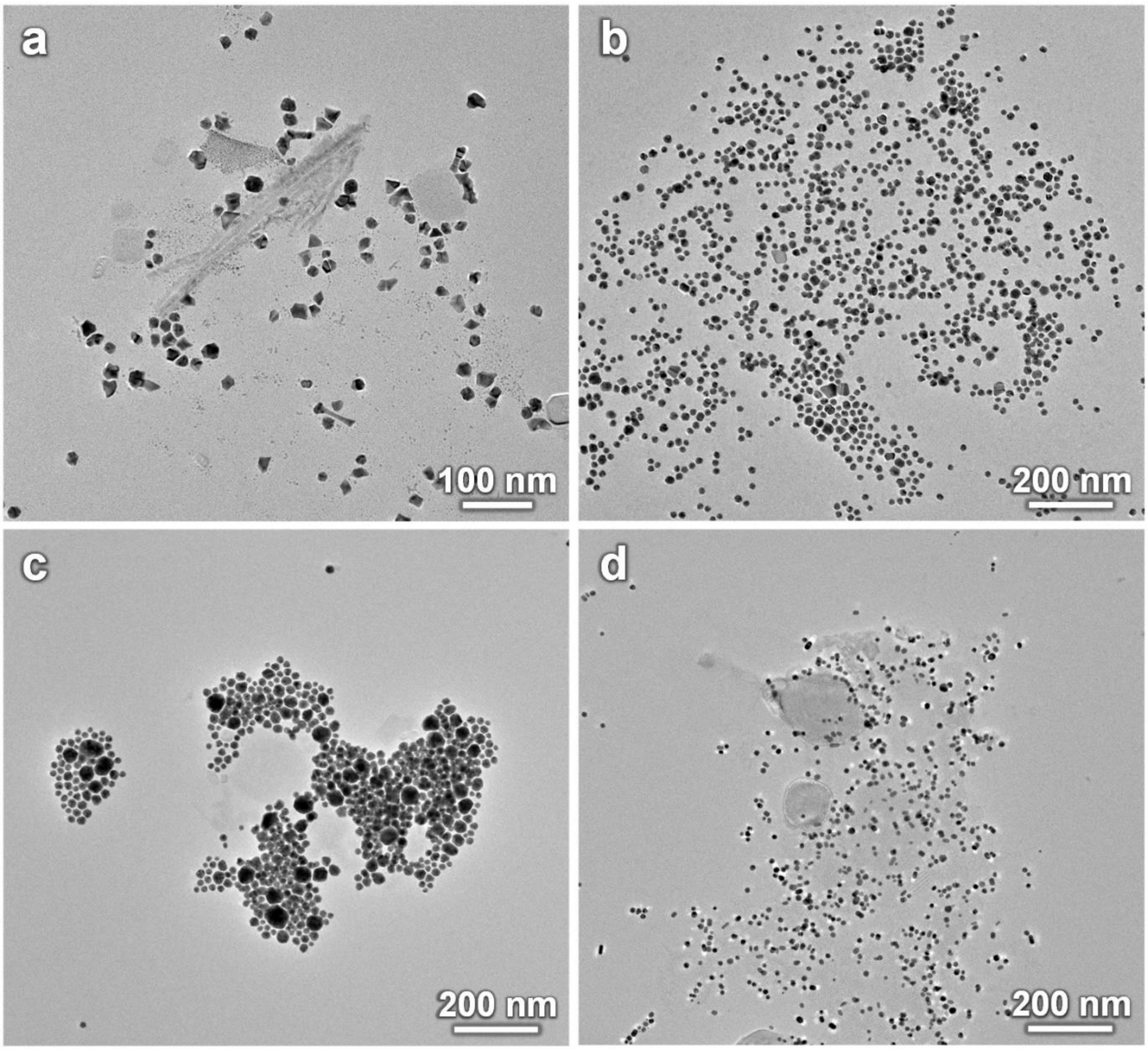

Supplementary Figure 4 | The effect of solvents. a-d, TEM images of Au nanostructures obtained by replacing the mixture of oleylamine and dodecylamine (v/v = 2/3) with (**a**) the pure oleylamine, (**b**) a mixture of oleylamine and octylamine (v/v = 2/3), (**c**) a mixture of oleylamine and hexylamine (v/v = 2/3), and (**d**) a mixture of oleylamine and dodecanol (v/v = 2/3).

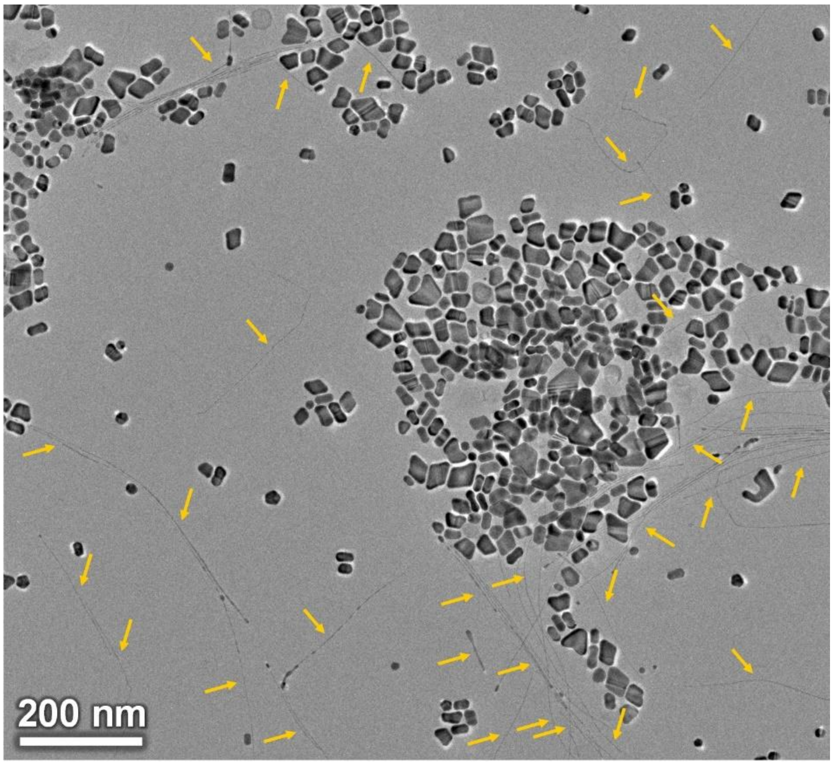

Supplementary Figure 5 | The effect of gas atmosphere. A typical TEM image of Au nanostructures obtained by changing the gas atmosphere from oxygen gas to air. The yellow arrows indicate the coexisting ultrathin Au nanowires, i.e., the by-product.

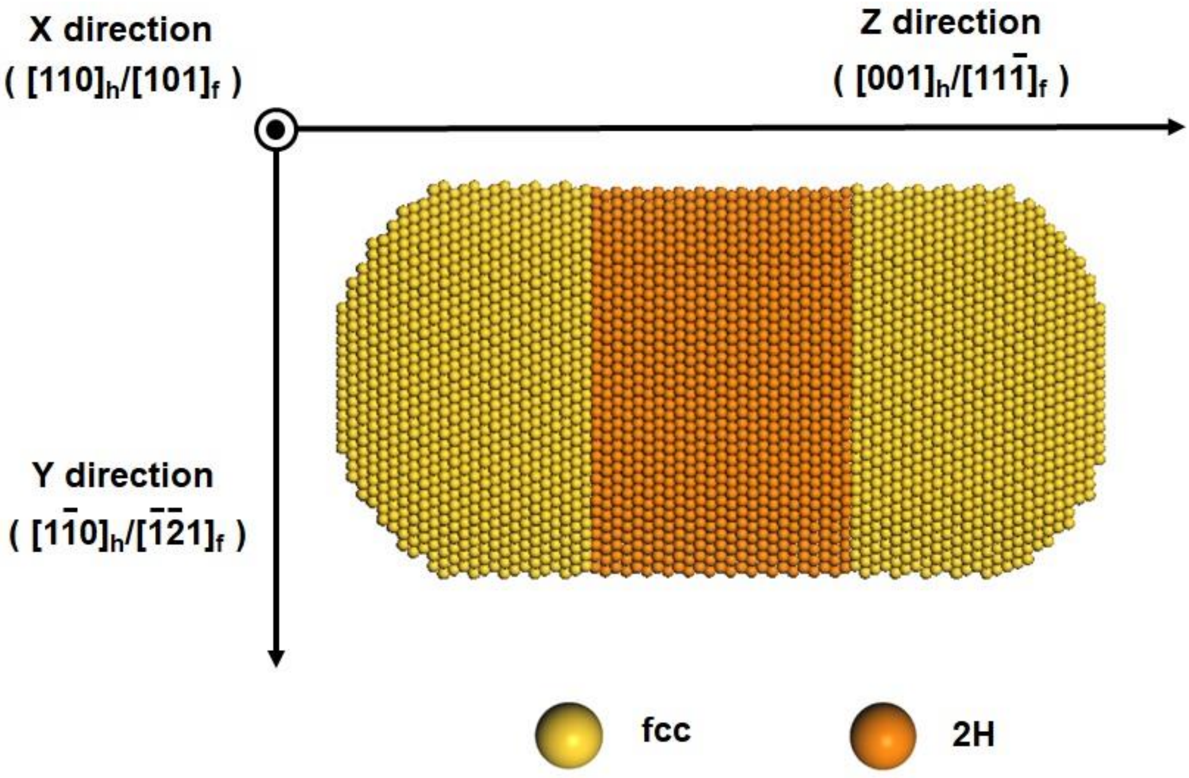

Supplementary Figure 6 | Schematic illustration for the view directions of fcc-2H-fcc Au NR. The atomic model of fcc-2H-fcc Au NR is taken from Fig. 1j. The X, Y and Z directions of Au NRs are parallel to the zone axes of $[110]_h/[101]_f$, $[1\bar{1}0]_h/[\bar{1}\bar{2}1]_f$ and $[001]_h/[11\bar{1}]_f$, respectively.

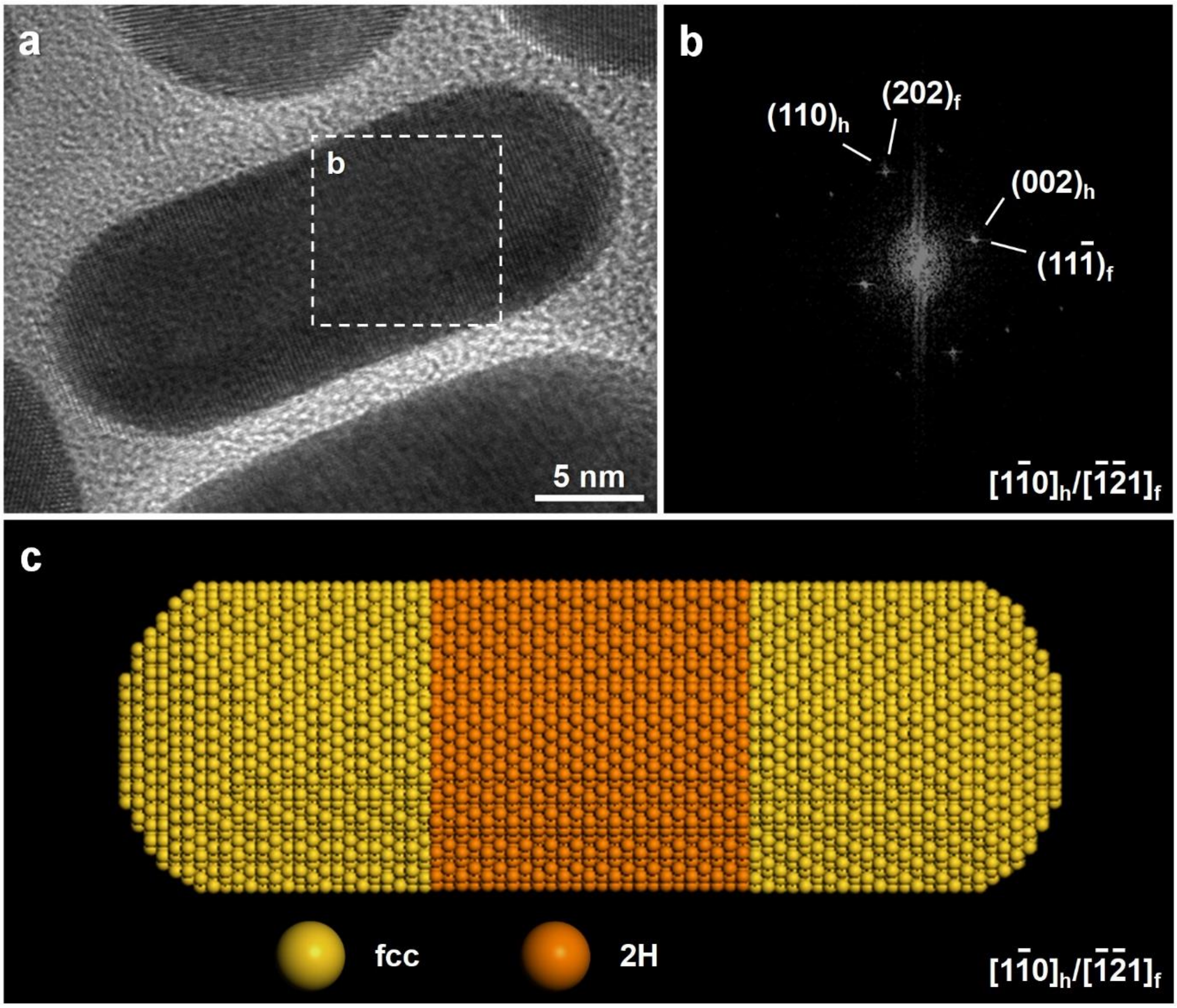

Supplementary Figure 7 | Structural characterization of fcc-2H-fcc Au NRs from the Y direction. **a**, HRTEM image taken from the Y direction of an Au NR. **b**, The corresponding FFT pattern of selected area in **a**, which matches well with the diffraction patterns of $[1\bar{1}0]_h$ and $[\bar{1}\bar{2}1]_f$ zone axes. **c**, An atomic model of the as-prepared heterophase fcc-2H-fcc Au NRs from the Y direction.

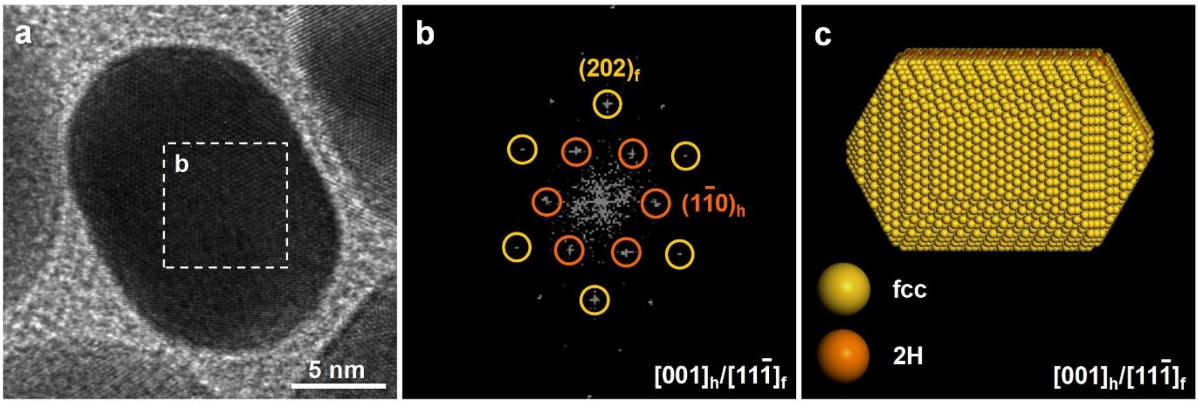

Supplementary Figure 8 | Structural characterization of fcc-2H-fcc Au NRs from the Z direction. **a**, HRTEM image taken from the Z direction of an Au NR. **b**, The corresponding FFT pattern of selected area in **a**, which shows two sets of diffraction spots belonging to the $(1\bar{1}0)_h$ and $(202)_f$ planes, respectively. It confirms the heterophase structure of the as-synthesized fcc-2H-fcc Au NRs. **c**, An atomic model of the as-prepared heterophase fcc-2H-fcc Au NRs from the Z direction.

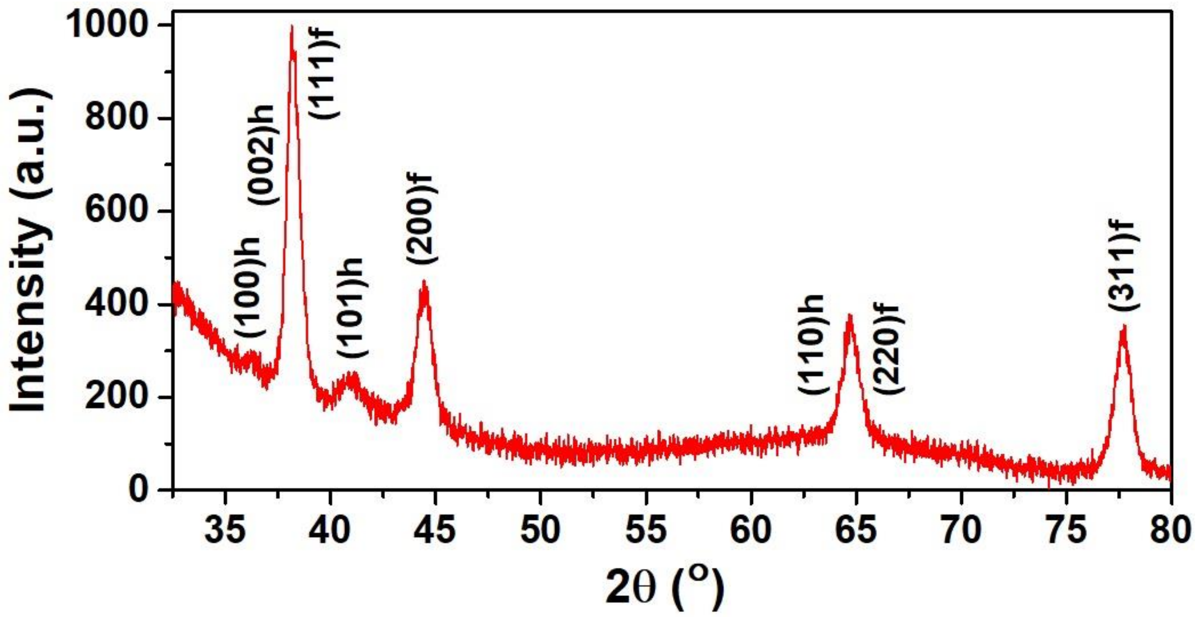

Supplementary Figure 9 | XRD characterization of fcc-2H-fcc Au NRs. The XRD pattern of fcc-2H-fcc Au NRs were collected in the θ/2θ mode. Two sets of diffraction peaks belonging to 2H and fcc phases are observed, as indicated in the XRD pattern, which further confirms the fcc-2H-fcc heterophase structure of the obtained Au NRs.

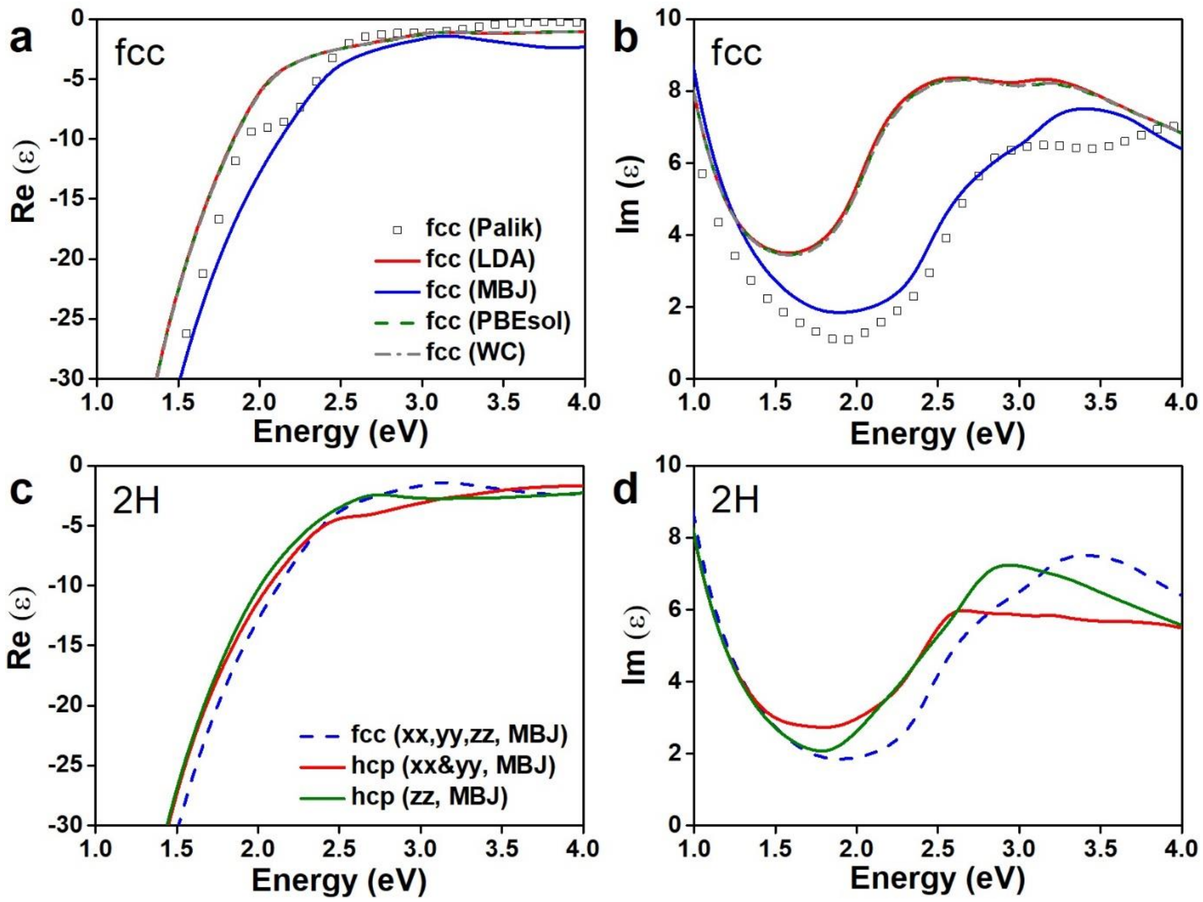

Supplementary Figure 10 | The calculations of dielectric permittivities for fcc Au and 2H Au. a-**d**, The computed complex dielectric permittivities of (**a**,**b**) fcc Au and (**c**,**d**) 2H Au using standard density functional theory (DFT) approaches. The computed functions for fcc Au are benchmarked against the experimentally measured permittivities from the Palik handbook (labeled as “Palik”). Note that the other abbreviations in this figure, i.e. “LDA”, “MBJ”, “PBEsol” and “WC”, stand for the “local density approximation”, “modified Becke-Johnson potential”, “revised Perdew-Burke-Ernzerh for solids generalized gradient approximation” and “Wu-Cohen generalized gradient approximation”, respectively.

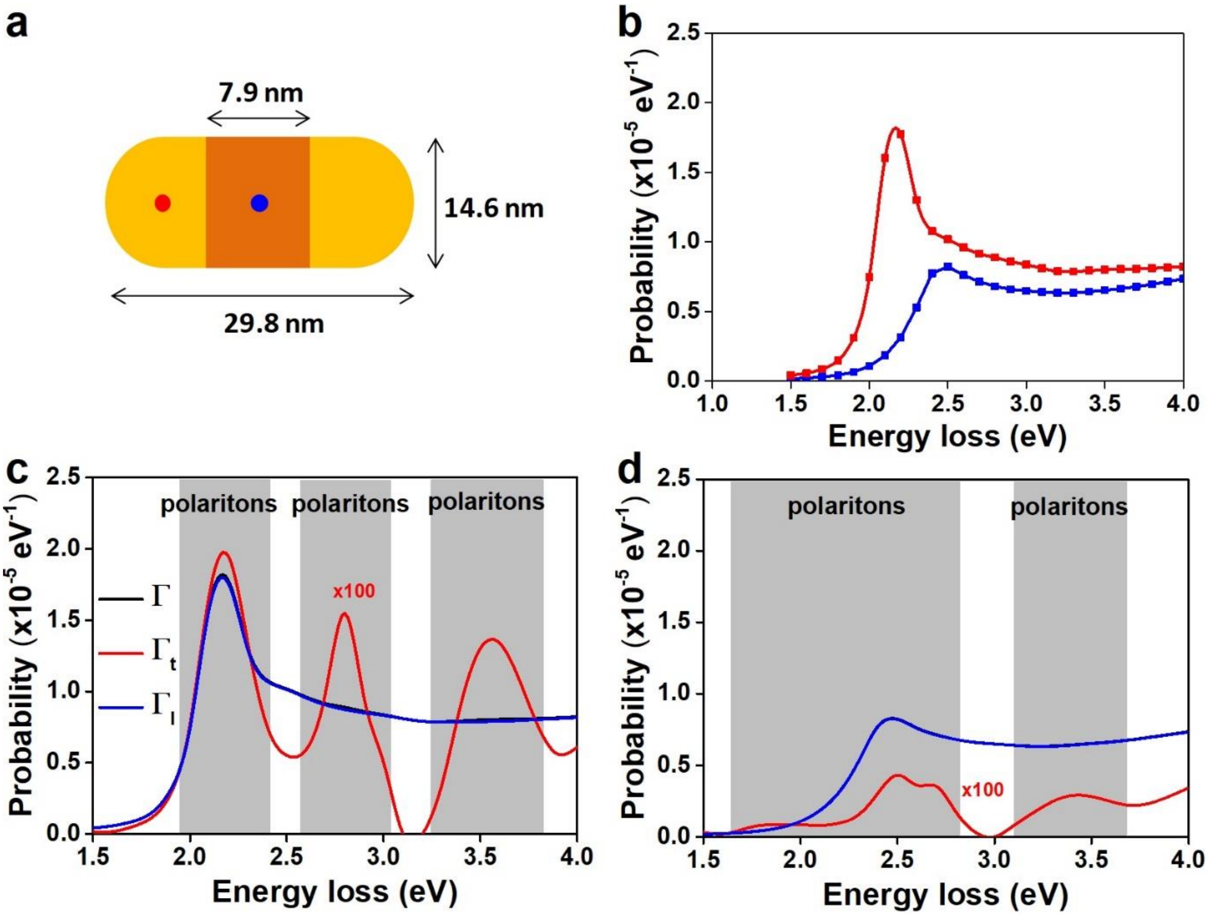

Supplementary Figure 11 | The Helmholtz decomposition of the EELS probability. a, The modelled heterophase fcc-2H-fcc Au NR. The model was built based on the Au NR shown in Fig. 2b. The length and width of the Au NR is 29.8 nm and 14.6 nm, respectively. The length of 2H phase in the middle of Au NR is measured to be 7.9 nm. **b**, The EELS spectra computed for two different positions of the E-beam shown by red and blue dots in **a**. **c**,**d**, Helmholtz decomposition of the EELS probability $\Gamma(\omega)$, which is obtained by locating the E-beam in the (**c**) end and (**d**) middle of Au NR, into loss contributions by transverse $\Gamma_t(\omega)$ and longitudinal $\Gamma_l(\omega)$ fields.

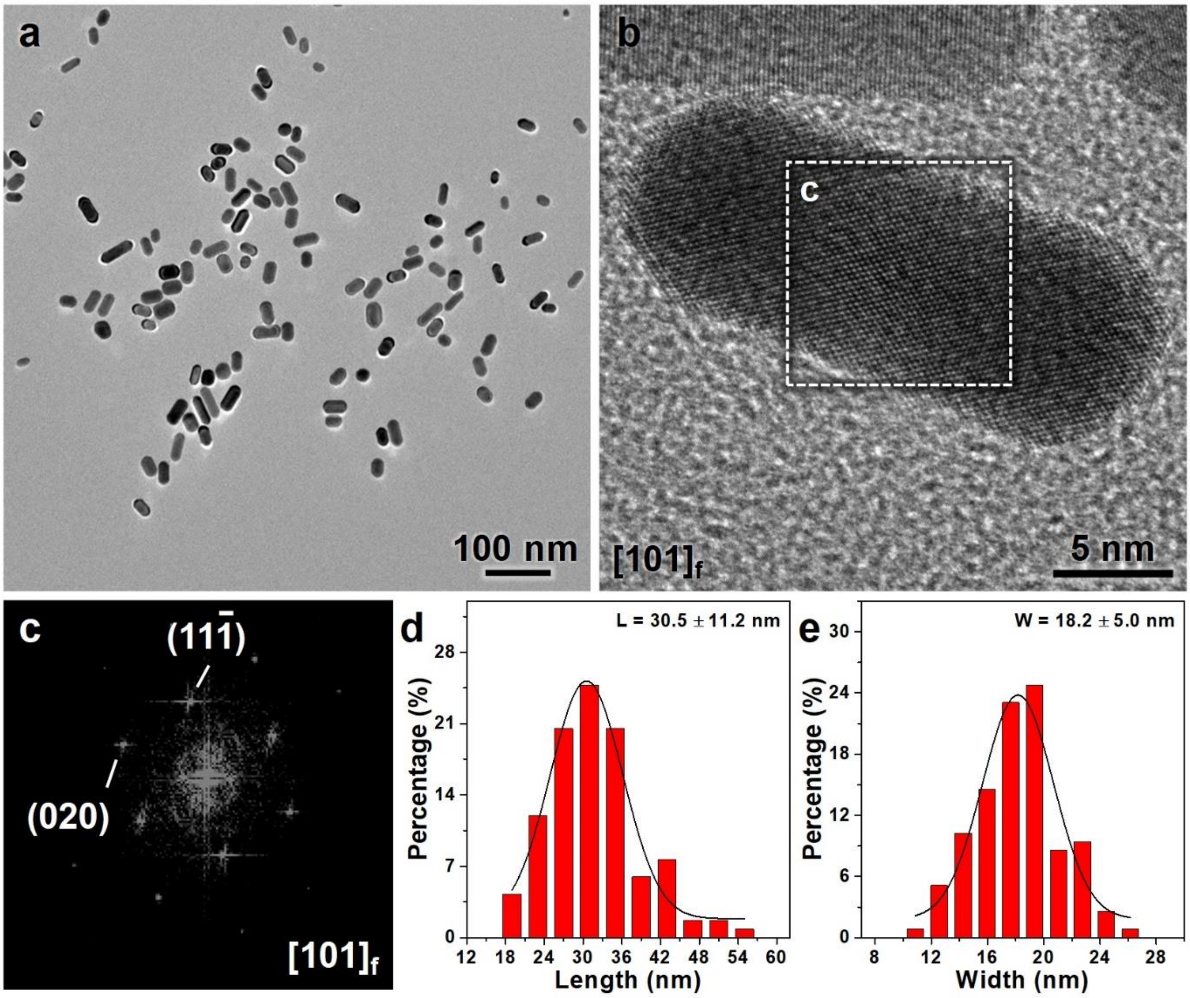

Supplementary Figure 12 | Structural characterization of fcc Au NRs. a,**b**, Low-magnification TEM (**a**) and HRTEM (**b**) images of fcc Au NRs. **c**, The corresponding FFT pattern of the selected area in **b**. **d**, Statistical analysis of the length of Au NRs measured by TEM, in which 30.5 nm is the average length and 11.2 nm is the standard deviation. **e**, Statistical analysis of the width of Au NRs measured by TEM, in which 18.2 nm is the average width and 5.0 nm is the standard deviation. The curves in **d** and **e** were obtained by fitting the data using a Gaussian function.

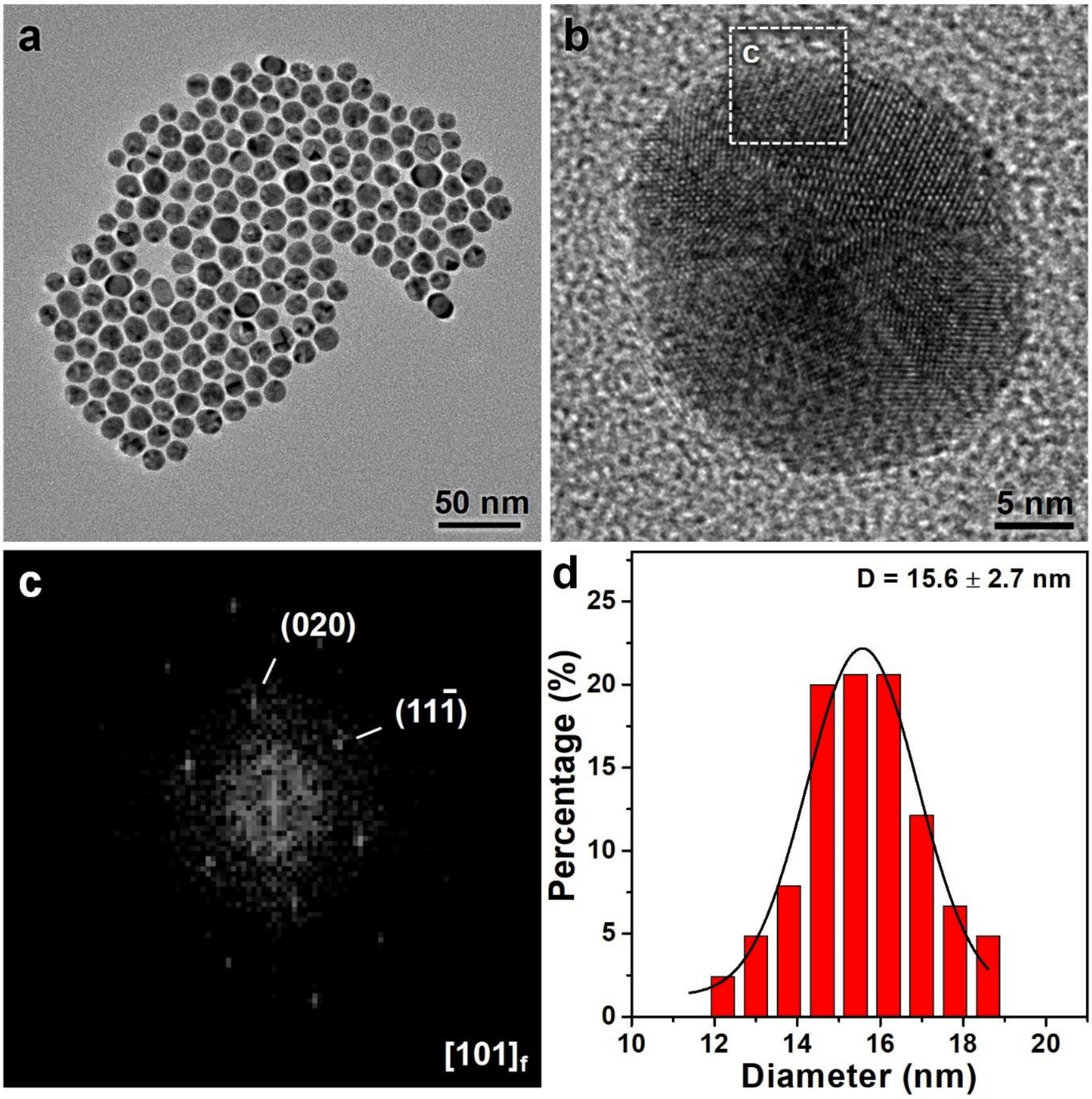

Supplementary Figure 13 | Structural characterization of fcc Au NPs. a,**b**, Low-magnification TEM (**a**) and HRTEM (**b**) images of fcc Au NPs. **c**, The corresponding FFT pattern of the selected area in **b**. **d**, Statistical analysis of the size of Au NPs measured by TEM, in which 15.6 nm is the average size and 2.7 nm is the standard deviation. The curve in **d** was obtained by fitting the data using a Gaussian function.

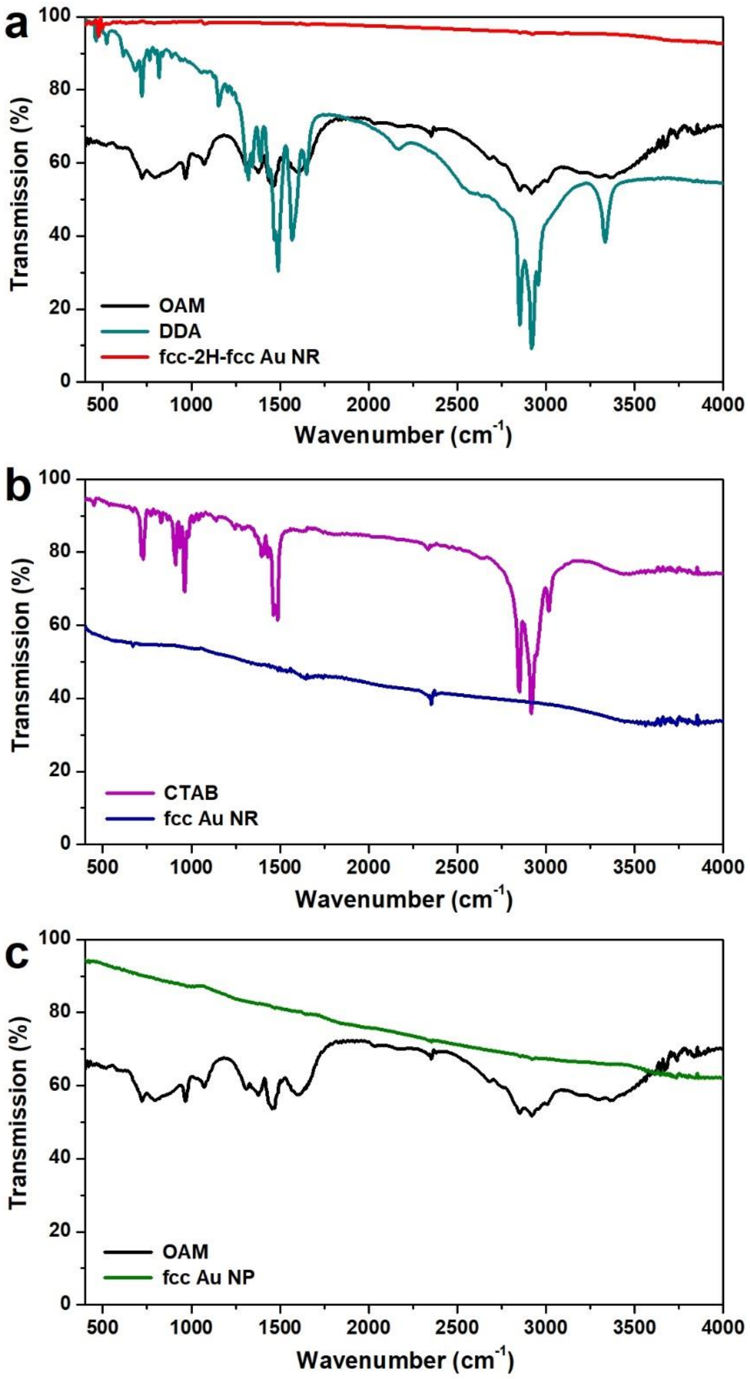

Supplementary Figure 14 | FTIR spectra of surface capping ligands and Au nanostructures after washing. a, FTIR spectra of oleylamine (OAM), dodecylamine (DDA) and the thoroughly washed fcc-2H-fcc Au NRs. **b**, FTIR spectra of cetyltrimethylammonium bromide (CTAB) and the thoroughly washed fcc Au NRs. **c**, FTIR spectra of OAM and the thoroughly washed fcc Au NPs. No obvious FTIR signals from surface capping ligands were observed after the Au nanostructures were thoroughly washed.

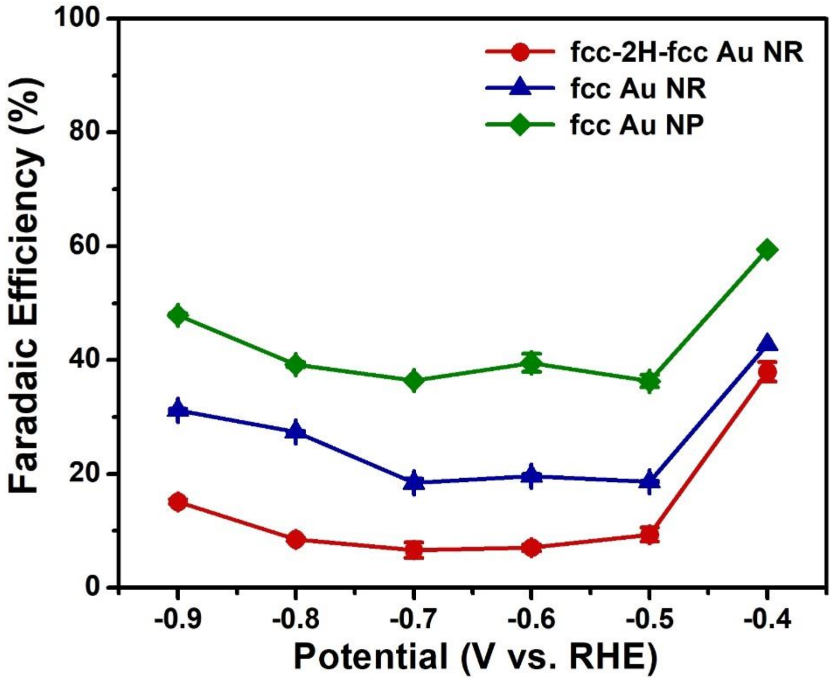

Supplementary Figure 15 | FEs of H_2. The FEs of H_2 for Au nanostructures, i.e. fcc-2H-fcc Au NRs, fcc Au NRs and fcc Au NPs at different applied potentials. Note that CO and H_2 are the only detectable products from the CO_2RR.

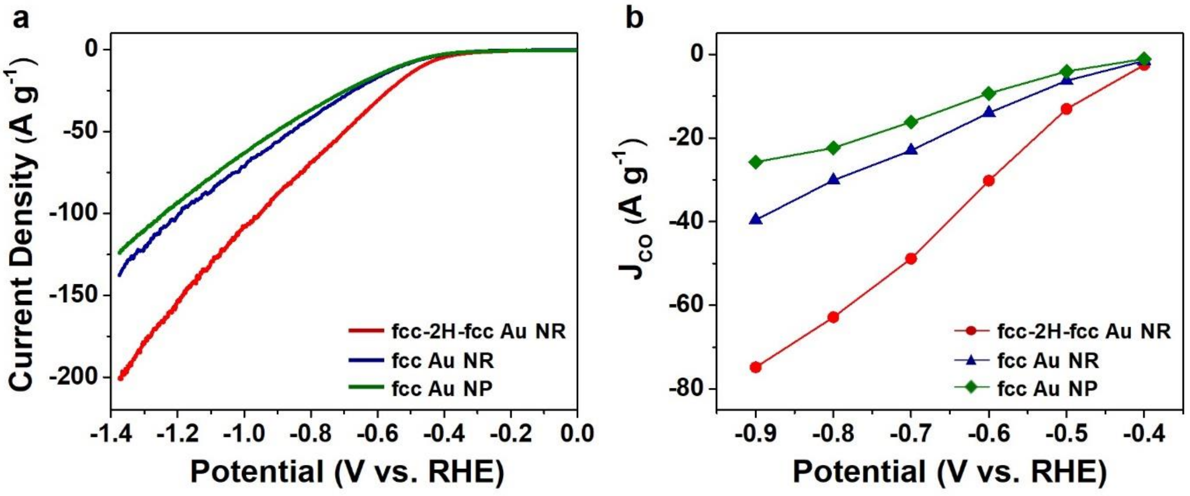

Supplementary Figure 16 | Mass activity. a,**b**, LSV curves (**a**) and CO partial current density (J_{CO}) (**b**) normalized by the mass of Au catalysts. Note that the total mass activity of fcc-2H-fcc Au NR is 30.8 A g^{-1}, and its partial mass activity for CO production is 30.2 A g^{-1}.

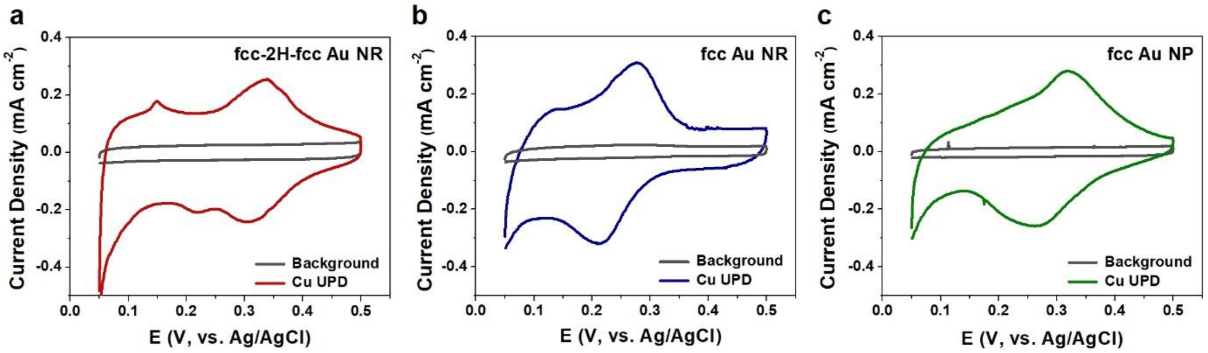

Supplementary Figure 17 | ECSA measurements. a-**c**, Cu underpotential deposition (UPD) and anodic stripping waves of (**a**) fcc-2H-fcc Au NR, (**b**) fcc Au NR and (**c**) fcc Au NP. The electrolyte solution is a mixture of $CuSO_4$ (0.1 M) and H_2SO_4 (0.5 M). The scan rate is 50 mV s^{-1}. ECSA was calculated by integrating the anodic peaks for Cu UPD stripping, and the baseline was determined in the absence of $CuSO_4$.

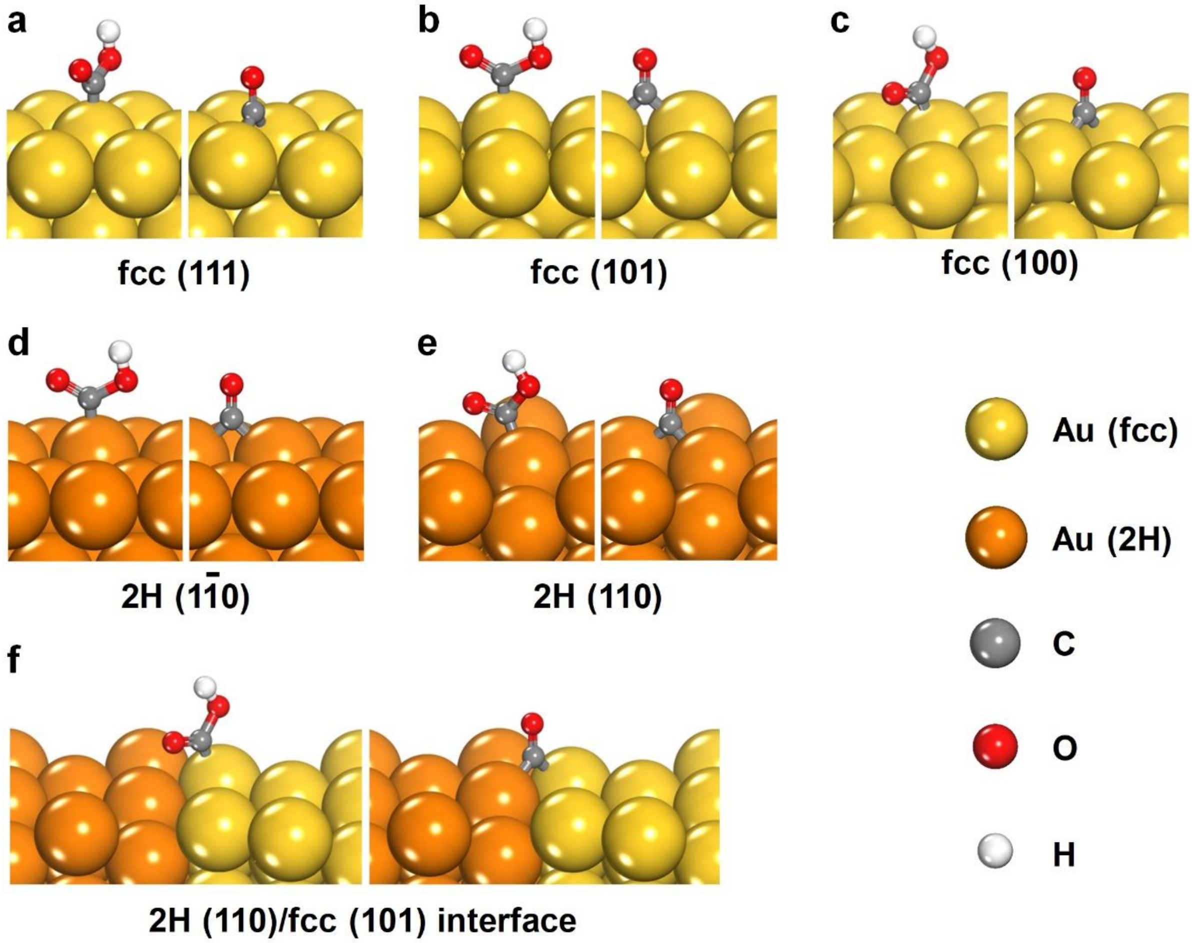

Supplementary Figure 18 | Theoretical models. a-**f**, Atomic models of *COOH (left panels) and *CO (right panels) adsorbed on various surfaces, including (**a**) fcc (111), (**b**) fcc (101), (**c**) fcc (100), (**d**) 2H (1$\bar{1}$0), (**e**) 2H (110), and (**f**) 2H (110)/fcc (101) interface.

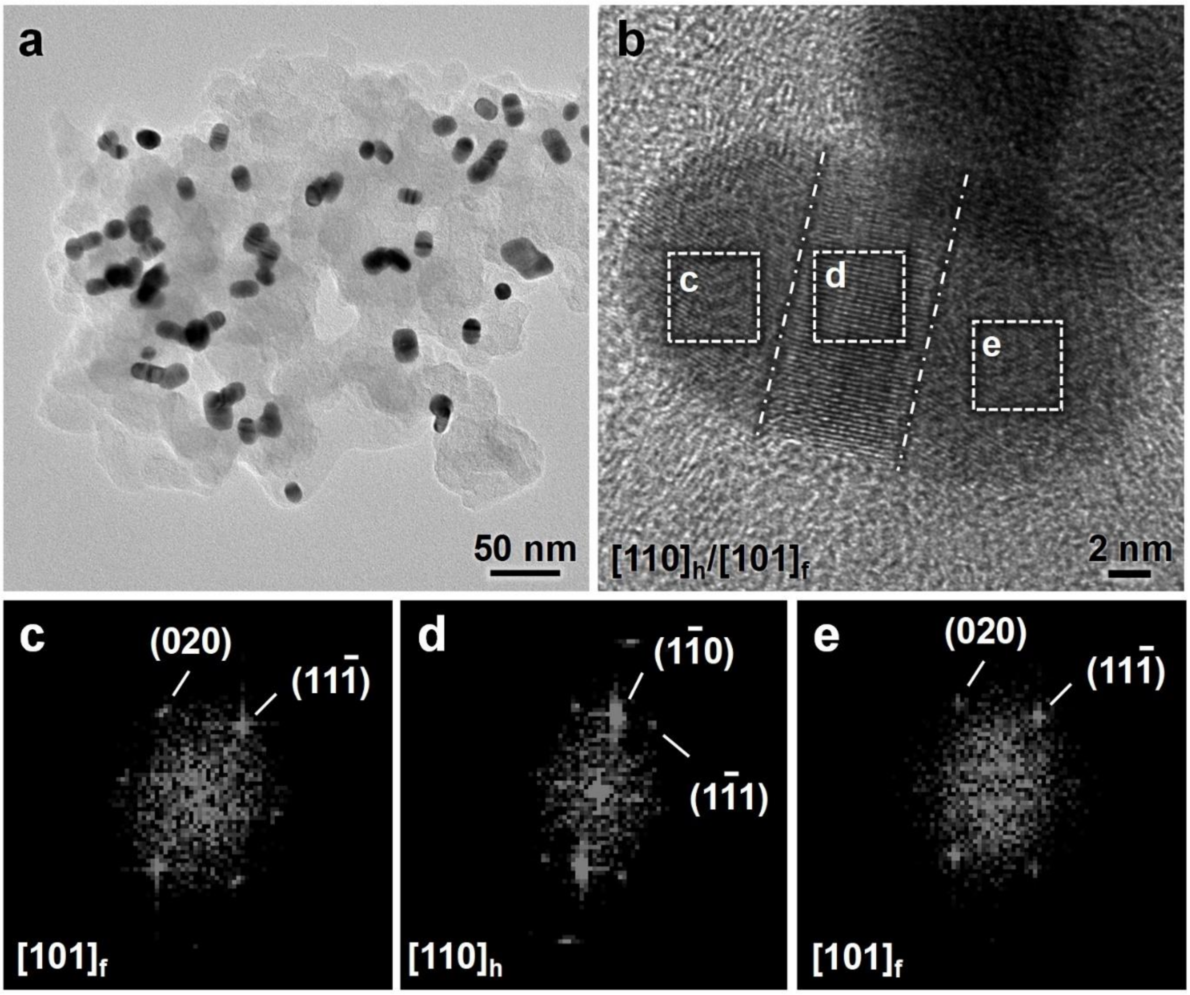

Supplementary Figure 19 | Structural characterization of fcc-2H-fcc Au NRs after the electrochemical durability measurement. a,**b**, TEM (**a**) and HRTEM (**b**) images of fcc-2H-fcc Au NRs after the stability test. The dashed-dotted white lines in **b** indicate the boundary between 2H and fcc phases. **c**-**e**, FFT patterns of the corresponding selected areas (**c**,**e**) in the two ends and (**d**) in the middle of the Au NR shown in **b**.

Supplementary Tables

**Supplementary Table 1 | ** Comparison of catalytic performance of noble metal catalysts in CO_2RR towards CO production in previous reports and this work.

Catalysts	Electrolytes	FE at -0.6 V / %	Mass activity at -0.6 V / A g^{-1}	Geometric current density at -0.6 V / mA cm^{-2}	Tafel slope / mV dec^{-1}	Refs.
**fcc-2H-fcc Au NR**	**0.5 M $KHCO_3$**	**98.2**	**30.8**	**8.18**	**64.9**	**This work**
8 nm Au NP	0.5 M $KHCO_3$	85	4.5	N. A.	N. A.	1
Ultrathin Au nanowire	0.5 M $KHCO_3$	90	6.3	N. A.	N. A.	2
Grain boundary-rich Au NP	0.5 M $NaHCO_3$	93	15	~ 0.9	N. A.	3
Au NP-CeO_x/C	0.1 M $KHCO_3$	70	7	3.6	N. A.	4
Tetradentate porphyrin-capped Au NP	0.5 M $KHCO_3$	93	N. A.	5.5	69	5
Thiolate-capped polycrystalline Au film	0.1 M $KHCO_3$	~ 33	N. A.	N. A.	N. A.	6
Carbene-functionalized Au NP	0.1 M $KHCO_3$	83	N. A.	~ 2	72	7
Au nanodendrite	0.2 M $KHCO_3$	95	N. A.	2.5	69	8
Au needle film	0.5 M $KHCO_3$	95	N. A.	80	42	9
Ag nanocoral film	0.1 M $KHCO_3$	95	N.A.	6.9	58.5	10
Ag NP	0.5 M $KHCO_3$	72	~ 8	~ 0.3	N. A.	11
Nanoporous Ag film	0.1 M KOH	90	N. A.	20	58	12
Triangular Ag nanoplates	0.1 M $KHCO_3$	~ 55	N. A.	0.5	153	13
Plasma-activated Ag foil	0.1 M $KHCO_3$	90	N. A.	2.5	N. A.	14

Oxide-derived Ag foil	0.2 M NaOH	80	N. A.	0.35	77	15
Oleylamine-Ag NP	0.5 M $KHCO_3$	89	12<br>(at -0.65V)	N. A.	N. A.	16
Pd@Pd_3Au_7 NP	0.5 M $KHCO_3$	97	0.04	5	68.6	17
19.4 nm Pd icosahedra	0.1 M $KHCO_3$	79	2.3	0.95	N. A.	18
3.7 nm Pd NP	0.1 M $KHCO_3$	83	N. A.	~ 1.8	N. A.	19

Supplementary Table 2 | List of zero point energy (E_{ZPE}), entropy (S), enthalpic temperature (T) and correction to the solvation effect (G_{sol}) to gas or liquid phase molecules and adsorbed species. The unit for energy is eV.

Species	E_{ZPE}	TS	$\int C_P dT$	G_{sol}
**CO_2 (g)**	0.31	0.65	0.10	0.00
***COOH**	0.62	0.18	0.09	-0.38
***CO**	0.19	0.15	0.08	-0.10

Supplementary Notes

Supplementary Note 1

To investigate the effect of Au precursor in the synthesis of heterophase fcc-2H-fcc Au NRs, with the same molar concentration, $HAuCl_4$, $AuCl_3 \cdot Py$, $AuCl_3$, and $Au(ac)_3$ were used to replace $KAuCl_4$, respectively, and the other experimental conditions were kept same. When $HAuCl_4$ was used, various kinds of Au nanostructures, such as Au nanoplates, Au nanowires and 5-fold twinned Au nanoparticles, were obtained (Supplementary Fig. 3a). Similar results were also observed when the Au precursor was changed from $KAuCl_4$ to $AuCl_3 \cdot Py$ or $AuCl_3$ (Supplementary Fig. 3b,c). However, when $KAuCl_4$ was replaced with $Au(ac)_3$, the obtained product was almost spherical Au nanoparticles (Supplementary Fig. 3d). The aforementioned experimental results indicate that the correct choice of Au precursor in our reaction is important for the successful synthesis of heterophase fcc-2H-fcc Au NRs.

Supplementary Note 2

To investigate the effect of solvent in the synthesis of heterophase fcc-2H-fcc Au NRs, the mixture of oleylamine and dodecylamine (v/v = 2/3) was replaced with the pure oleylamine, a mixture of oleylamine and octylamine (v/v = 2/3), a mixture of oleylamine and hexylamine (v/v = 2/3), and a mixture of oleylamine and dodecanol (v/v = 2/3), respectively, and the other experimental conditions were kept same. When the pure oleylamine was used as the solvent, irregular-shaped Au nanoparticles were obtained (Supplementary Fig. 4a). When the mixture of oleylamine and octylamine or the mixture of oleylamine and hexylamine was used as the solvent, spherical Au nanoparticles were obtained (Supplementary Fig. 4b,c). Moreover, when the mixture of oleylamine and dodecanol was used as the solvent, spherical Au nanoparticles together with little amount of Au nanorods were obtained (Supplementary Fig. 4d). The aforementioned experimental results indicate that the correct choice of solvent in the reaction is important for the successful synthesis of heterophase fcc-2H-fcc Au NRs.

Supplementary Note 3

To investigate the effect of gas atmosphere in the synthesis of heterophase fcc-2H-fcc Au NRs, air was used to replace the oxygen gas, and the other experimental conditions were kept same. When the reaction was conducted in air, besides non-uniform Au nanorods with large size distribution, irregular-shaped Au nanostructures and ultrathin Au nanowires were also obtained (Supplementary Fig. 5), indicating that the correct choice of gas atmosphere in the reaction is important for the successful synthesis of heterophase fcc-2H-fcc Au NRs.

Supplementary References

1 Zhu, W. *et al.* Monodisperse Au nanoparticles for selective electrocatalytic reduction of CO_2 to CO. *J. Am. Chem. Soc.* **135**, 16833-16836 (2013).

2 Zhu, W. *et al.* Active and selective conversion of CO_2 to CO on ultrathin Au nanowires. *J. Am. Chem. Soc.* **136**, 16132-16135 (2014).

3 Feng, X., Jiang, K., Fan, S. & Kanan, M. W. Grain-boundary-dependent CO_2 electroreduction activity. *J. Am. Chem. Soc.* **137**, 4606-4609 (2015).

4 Gao, D. *et al.* Enhancing CO_2 electroreduction with the metal–oxide interface. *J. Am. Chem. Soc.* **139**, 5652-5655 (2017).

5 Cao, Z. *et al.* Chelating N-heterocyclic carbene ligands enable tuning of electrocatalytic CO_2 reduction to formate and carbon monoxide: surface organometallic chemistry. *Angew. Chem. Int. Ed.* **57**, 4981-4985 (2018).

6 Fang, Y. & Flake, J. C. Electrochemical reduction of CO_2 at functionalized Au electrodes. *J. Am. Chem. Soc.* **139**, 3399-3405 (2017).

7 Cao, Z. *et al.* A molecular surface functionalization approach to tuning nanoparticle electrocatalysts for carbon dioxide reduction. *J. Am. Chem. Soc.* **138**, 8120-8125 (2016).

8 Kim, J. *et al.* Morphology-controlled Au nanostructures for efficient and selective electrochemical CO_2 reduction. *J. Mater. Chem. A* **6**, 5119-5128 (2018).

9 Liu, M. *et al.* Enhanced electrocatalytic CO_2 reduction via field-induced reagent concentration. *Nature* **537**, 382-386 (2016).

10 Hsieh, Y.-C., Senanayake, S. D., Zhang, Y., Xu, W. & Polyansky, D. E. Effect of chloride anions on the synthesis and enhanced catalytic activity of silver nanocoral electrodes for CO_2 electroreduction. *ACS Catal.* **5**, 5349-5356 (2015).

11 Kim, C. *et al.* Achieving selective and efficient electrocatalytic activity for CO_2 reduction using immobilized silver nanoparticles. *J. Am. Chem. Soc.* **137**, 13844-13850 (2015).

12 Lu, Q. *et al.* A selective and efficient electrocatalyst for carbon dioxide reduction. *Nat. Commun.* **5**, 3242 (2014).

13 Liu, S. *et al.* Shape-dependent electrocatalytic reduction of CO_2 to CO on triangular silver nanoplates. *J. Am. Chem. Soc.* **139**, 2160-2163 (2017).

14 Mistry, H. *et al.* Enhanced carbon dioxide electroreduction to carbon monoxide over defect-rich plasma-activated silver catalysts. *Angew. Chem. Int. Ed.* **56**, 11394-11398 (2017).

15 Ma, M., Trześniewski, B. J., Xie, J. & Smith, W. A. Selective and efficient reduction of carbon dioxide to carbon monoxide on oxide-derived nanostructured silver electrocatalysts. *Angew. Chem. Int. Ed.* **55**, 9748-9752 (2016).

16 Kim, C. *et al.* Insight into electrochemical CO_2 reduction on surface-molecule-mediated Ag nanoparticles. *ACS Catal.* **7**, 779-785 (2017).

17 Yuan, X. *et al.* Ultrathin Pd–Au shells with controllable alloying degree on Pd nanocubes toward carbon dioxide reduction. *J. Am. Chem. Soc.* **141**, 4791-4794 (2019).

18 Huang, H. *et al.* Understanding of Strain effects in the electrochemical reduction of CO_2: using Pd nanostructures as an ideal platform. *Angew. Chem. Int. Ed.* **56**, 3594-3598 (2017).

19 Gao, D. *et al.* Size-dependent electrocatalytic reduction of CO_2 over Pd nanoparticles. *J. Am. Chem. Soc.* **137**, 4288-4291 (2015).
